# Tumor residue in patients with stage II–IVA nasopharyngeal carcinoma who received intensity-modulated radiation therapy: development and validation of a prediction nomogram integrating postradiotherapy plasma Epstein–Barr virus deoxyribonucleic acid, clinical stage, and radiotherapy dose

**DOI:** 10.1186/s12885-023-10827-0

**Published:** 2023-05-06

**Authors:** Ying-Ying Huang, Jia-Yu Zhou, Ze-Jiang Zhan, Liang-Ru Ke, Wei-Xiong Xia, Xun Cao, Zhuo-Chen Cai, Ying Deng, Xi Chen, Lu-Lu Zhang, Hao-Yang Huang, Xiang Guo, Xing Lv

**Affiliations:** 1 State Key Laboratory of Oncology in South China, Collaborative Innovation Center for Cancer Medicine, Guangdong Key Laboratory of Nasopharyngeal Carcinoma Diagnosis and Therapy, Sun Yat-Sen University Cancer Center, 651 Dongfeng East Road, Guangzhou, 510060 People’s Republic of China; 2 Department of Nasopharyngeal Carcinoma, Sun Yat-Sen University Cancer Center, 651 Dongfeng East Road, Guangzhou, 510060 People’s Republic of China; 3 Department of Medical Imaging, Sun Yat-Sen University Cancer Center, 651 Dongfeng East Road, Guangzhou, 510060 People’s Republic of China; 4 Department of Critical Care Medicine, Sun Yat-Sen University Cancer Center, 651 Dongfeng East Road, Guangzhou, 510060 People’s Republic of China

**Keywords:** Nasopharyngeal carcinoma, Intensity-modulated radiotherapy, Tumor residue, Epstein–Barr virus deoxyribonucleic acid, Prognostic value, Prediction nomogram

## Abstract

**Background:**

To develop and validate a predictive nomogram for tumor residue 3–6 months after treatment based on postradiotherapy plasma Epstein–Barr virus (EBV) deoxyribonucleic acid (DNA), clinical stage, and radiotherapy (RT) dose in patients with stage II–IVA nasopharyngeal carcinoma (NPC) treated with intensity-modulated radiation therapy (IMRT).

**Methods:**

In this retrospective study, 1050 eligible patients with stage II–IVA NPC, who completed curative IMRT and underwent pretreatment and postradiotherapy (-7 to +28 days after IMRT) EBV DNA testing, were enrolled from 2012 to 2017. The prognostic value of the residue was explored using Cox regression analysis in patients (*n*=1050). A nomogram for predicting tumor residues after 3–6 months was developed using logistic regression analyses in the development cohort (*n*=736) and validated in an internal cohort (*n*=314).

**Results:**

Tumor residue was an independent inferior prognostic factor for 5-year overall survival, progression-free survival, locoregional recurrence-free survival and distant metastasis-free survival (all *P*<0.001). A prediction nomogram based on postradiotherapy plasma EBV DNA level (0 vs. 1–499 vs. ≥500 copies/ml), clinical stage (II vs. III vs. IVA), and RT dose (68.00–69.96 vs. 70.00–74.00 Gy) estimated the probability of residue development. The nomogram showed better discrimination (area under the curve (AUC): 0.752) than either the clinical stage (0.659) or postradiotherapy EBV DNA level (0.627) alone in the development and validation cohorts (AUC: 0.728).

**Conclusions:**

We developed and validated a nomogram model integrating clinical characteristics at the end of IMRT for predicting whether tumor will residue or not after 3–6 months. Thus, high-risk NPC patients who might benefit from immediate additional intervention could be identified by the model, and the probability of residue can be reduced in the future.

**Supplementary Information:**

The online version contains supplementary material available at 10.1186/s12885-023-10827-0.

## Background

Nasopharyngeal carcinoma (NPC) is malignant disease of multidimensional spatiotemporal unity of ecology and evolution, often manifesting with invasive growth at the primary site and metastatic cervical lymph node(s) [1, 2]. Although the implementation of intensity-modulated radiotherapy (IMRT) has attained satisfactory tumor control and survival benefits in NPC [3–5]. Intertumor and intratumor heterogeneities in the ecosystem of NPC have caused diverse response patterns among patients [1].

Due to the different regression rate in tumor and disturbance of (chemo) radiotherapy-induced inflammation, 12 weeks after the completion of radiotherapy (RT) is proposed as a proper time point for initial evaluation of total tumor control [6, 7]. By then, inflammation would have largely resolved, most tumors would have regressed, and delayed tumor regression within 12 weeks would not impair overall control [8, 9]. Beyond this window time (12 weeks), the incidence of residue increased [8]. Reportedly, 3–13% of patients had persistent disease after 12 weeks and were diagnosed as tumor residue [10, 11]. Residual tumor stem cells have evolved aggressively and grow more rapidly during treatment cessation [12]. Thus, most patients with residual disease ultimately develop disease failure [10, 11].

Residue may be a reflection of treatment insensitivity, inadequate RT dose, or geographic miss in irradiated field [12]. Timely additional treatment (including surgery, boost radiation, and chemotherapy) at the end of RT may enhance or strengthen the curative effect and reduce the probability of residue after 3–6 months [13–15]. Controversially, the blind administration of additional intervention at the end of RT may be surplus for some patients whose tumor may spontaneous regressed. However, there is no sound tool at the end of RT to predict whether tumor will residue or not after observation for 3–6 months.

In NPC, Epstein–Barr virus (EBV) deoxyribonucleic acid (DNA) serves as a liquid circulating biomarker that reflects the tumor burden [16]. Reportedly, postradiotherapy EBV DNA has an even stronger association with recurrences and poor prognosis in NPC [17, 18]. Elevated plasma EBV DNA level has been shown to predate clinical recurrence by 3 to 7 months, which may present a biomarker of subclinical residual disease [18, 19]. But its predictive value on residue is unexcavated.

Here, we hypothesized that the probability of tumor residues could be reduced through immediate additional intervention at the end of IMRT by leveraging a predictive tool. In this study, we aimed to develop and validate a nomogram model integrating clinical characteristics at the end of IMRT for predicting whether tumor will residue or not after 3–6 months. Thus, NPC patients with a high risk of residue event might benefit from immediate additional intervention. Meanwhile, low-risk patients who will have tumor complete regressed after 3 months will be spared from overtreatment.

## Methods

### Patients

Between January 2012 and December 2017, patients at Sun Yat-sen University Cancer Center who fulfilled the inclusion criteria were enrolled. The inclusion criteria were as follows: (1) histologically confirmed non-metastatic NPC without previous or concurrent malignant disease; (2) age ≥ 18 years; (3) receipt of curative IMRT for the entire course without interruption; (4) available information on pretreatment and postradiotherapy (-7 to +28 days) plasma EBV DNA levels; (5) regular follow-up with complete posttreatment examination, including nasopharyngoscopy, magnetic resonance imaging (MRI) of the nasopharynx and neck, etc., until the first documentation of disease failure or death; and (6) no evidence of distant metastasis on chest scan (x-ray or computed tomography [CT]), liver scan (external ultrasonography or CT), bone scan, or 18F-fluorodeoxyglucose positron emission tomography-computed tomography (18F-FDG PET-CT) up to 6 months postradiotherapy.

A total of 1080 patients were included in this study. All patients were restaged according to the 8^th^ edition of the International Union for Cancer Control/American Joint Committee on Cancer staging system [20]. Because patients with stage I disease obtained extremely satisfactory disease control, adjuvant therapy is not recommended for patients with stage I disease [21–23]. Also, none of the 30 patients with stage I disease have residual disease. Therefore, patients with stage I disease was excluded from this study. Eventually, 1050 patients with stage II–IVA NPC were enrolled. The Institutional Review Board of Sun Yat-sen University Cancer Center approved this study (B2021-215–01).

### Plasma EBV DNA measurement

Plasma EBV DNA levels were measured using a quantitative polymerase chain reaction assay targeting the BamHI-W region of the EBV genome [24, 25]. The EBV DNA level was measured within 28 days before treatment (pretreatment) and -7 to +28 days after completion of IMRT (postradiotherapy).

### Diagnostic criteria of residue

Residue is defined as the confirmation of disease occurring within 6 months after treatment. When disease is found after these 6 months, provided that previous complete remission was seen, it is defined as recurrence [26]. Residue was firstly found by physical examination, nasopharyngoscopy or imaging modality (e.g., MRI, CT). By histopathological or cytological biopsy, or pathological examination of surgery-resected lesion, the lesion contained any component with cancerous cells or tissue originating from the primary NPC is diagnosed as residue. For lesions that were not accessible (e.g., skull base, intracranial residue), the diagnoses were made based on 18F-FDG PET/CT in consensus by two nuclear medicine physicians (each with 5 years of experience in PET/CT) using a GE Xeleris workstation. Image interpretation was based on visual evaluations. Any focus of FDG uptake greater than the surrounding background and not attributable to normal FDG biodistribution was assessed. The intensity of FDG uptake was graded using the five-point scale proposed by Ng et al. [27, 28]. The probability of residual tumor was graded by using a five-point scoring system (0 = no lesion, 1 = definitely benign, 2 = probably benign, 3 = probably malignant, and 4 = definitely malignant). Grade 3 and grade 4 were both considered to be positive results. The types of residue were as follows: local residue: residue in the nasopharynx, and/or extension to the oropharynx, nasal cavity, parapharyngeal space, adjacent soft tissue, and/or infiltration of bony structures at the skull base, cervical vertebra, pterygoid structures, paranasal sinuses, and intracranial extension; regional residue: residual tumor in retropharyngeal lymph nodes and/or cervical lymph nodes; and locoregional residue: both local and regional residues.

### Therapeutic regimens

All patients received radical IMRT as the primary treatment. Details regarding IMRT techniques have been described in previous studies [29]. Target volume delineation was performed according to the International Commission Radiological Units guidelines. Doses to critical normal structures and plan evaluations were directed according to the Radiation Therapy Oncology Group guidelines. The prescribed dose of 68.00–74.00 Gy was delivered in 30–33 fractions for the primary tumor and any involved lymph nodes. Platinum-based chemotherapy, including concurrent chemoradiotherapy every 3 weeks for 2–3 cycles or weekly for 6–7 cycles, and neoadjuvant chemotherapy every 3 weeks for 2–4 cycles, were implemented at the physician’s discretion depending on the patient’s physical status and disease stage.

### Follow-up

Patients were assessed every 3 months during the first 3 years, every 6 months during the next 2 years, and annually thereafter. The last follow-up date was 31 May 2022. From the start of treatment to the date of death from any cause, first occurrence of treatment failure or death, first occurrence of locoregional failure or death, and first remote failure or death were measured as overall survival (OS), progression-free survival (PFS), locoregional relapse-free survival (LRRFS), and distant metastasis-free survival (DMFS), respectively.

### Statistical analyses

Categorical variables were presented as frequencies and percentages. Continuous variables were described using the median and interquartile range. Pearson’s chi-square test or Fisher’s exact test were used to assess categorical variables between the groups. Differences in non-normally distributed variables between the groups were examined using the Mann–Whitney *U* test. Survival rates were calculated using the Kaplan–Meier method and compared using the log-rank test. Univariate and multivariate analyses with the Cox proportional hazards model were used to identify significant independent prognostic factors using forward elimination. The optimal threshold analysis of the pretreatment and postradiotherapy EBV DNA levels in predicting residue was conducted based on a receiver operating characteristic (ROC) curve. Logistic regression analysis was performed to identify variables associated with the residue. Variables achieving a significance level of *P*<0.05 in the univariate analysis were subjected to multivariate logistic regression analysis. The discriminating ability of a model was described by the area under the curve (AUC), and the calibration was evaluated by constructing a calibration curve. The clinical usefulness of the nomogram was evaluated using decision curve analysis (DCA). The performance of the models was evaluated by calculating the AUC, which was calculated and compared using the method suggested by Delong et al. [30] using MedCalc 19.6.4 (MedCalc Software Ltd.). The optimal cut-off value of the points predicted by the nomogram was selected based on the ROC curve in the development cohort, and the same cut-off value was applied to the validation cohort. All analyses were performed using SPSS software (version 24.0; IBM Corp., Chicago, IL, USA) and R-4.2.0 (http://www.R-project.org/). A two-sided *P*<0.05 was considered statistically significant.

## Results

### Patients’ characteristics

**Fig. 1:**
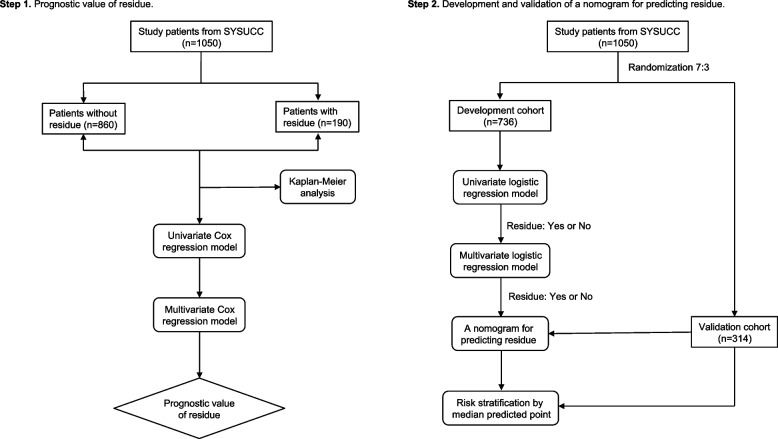
The flowchart of the study

The flowchart of the study is presented in Fig. 1. From January 2012 to December 2017, 1050 patients with stage II–IVA NPC were recruited for this study. Among them, 190 (18.1%) patients were diagnosed with residue. In particular, the residue type was mainly regional residue (111/190, 58.4%), while local residue accounted for 26.3% (50/190) and locoregional residue for 15.3% (29/190). The characteristics of patients with and without residue are listed in Table 1. Between the two groups, residue was significantly associated with primary tumor stage, metastatic lymph node stage, and clinical stage, especially in those with advanced disease (*P*<0.001). As for plasma EBV DNA levels, there was no difference between the two groups in terms of pretreatment EBV DNA levels (*P*= 0.161). However, a higher proportion of patients with residue tended to have detectable postradiotherapy EBV DNA levels (30.5% vs. 6.2%, *P*<0.001). Moreover, an elevated level of postradiotherapy plasma EBV DNA was observed in those with residue (median:1085 vs. 336 copies/ml, *P*=0.015). In a median follow-up duration of 63.9 months (range, 7.7–112.2 months), 101 (9.6%) patients died, and 265 (25.2%) patients experienced disease failure (locoregional failure: 251, 23.9%; distant metastasis: 60, 5.7%).

**Table 1:** Clinical characteristics between patients with and without tumor residue

**Characteristics**	**With Residue** **No. (%)**	**Without Residue** **No. (%)**	***P***^e^
**Total**	*n*= 190	*n*= 860	
**Sex**			0.825
Male	145 (76.3)	647 (75.2)	
Female	45 (23.7)	213 (24.8)	
**Age, years**			0.093
<45	99 (52.1)	508 (59.1)	
≥45	91 (47.9)	352 (40.9)	
**Histological type**^a^			1.000
I&II	2 (1.1)	10 (1.2)	
III	188 (98.9)	850 (98.8)	
**T category**^b^			<0.001
T2	38 (20.0)	234 (27.2)	
T3	94 (49.5)	489 (56.9)	
T4	58 (30.5)	137 (15.9)	
**N category**^b^			<0.001
N0	9 (4.7)	61 (7.1)	
N1	63 (33.2)	366 (42.6)	
N2	60 (31.6)	315 (36.6)	
N3	58 (30.5)	118 (13.7)	
**AJCC TNM stage**^b^			<0.001
II	11 (5.8)	145 (16.9)	
III	72 (37.9)	472 (54.9)	
IVA	107 (56.3)	243 (28.3)	
**Pretreatment EBV DNA, copies/ml**			<0.001
0	29 (15.3)	246 (28.6)	
1–4999	77 (40.5)	323 (37.6)	
≥5000	84 (44.2)	291 (33.8)	
No of patients (%) with detectable pretreatment EBV DNA (>0), copies/ml	161 (84.7)	614 (71.4)	
Median (IQR)	6100 (1235–28200)	4450 (1180–17300)	0.161
**Treatment modality**			<0.001
RT	7 (3.7)	84 (9.8)	
CCRT	68 (35.8)	401 (46.6)	
NACT+CCRT	115 (60.5)	375 (43.6)	
**RT dose to nasopharynx ± metastatic cervical lymph node(s), Gy**			0.009
68.00–69.96	108 (56.8)	396 (46.0)	
70.00–74.00	82 (43.2)	464 (54.0)	
Median (IQR)	69.96 (69.90–70.08)	70.00 (69.90–70.00)	0.358
**Postradiotherapy EBV DNA, copies/ml**			<0.001
0	132 (69.5)	807 (93.8)	
1–499	21 (11.0)	28 (3.3)	
≥500	37 (19.5)	25 (2.9)	
No of patients (%) with detectable postradiotherapy EBV DNA (>0), copies/ml	58 (30.5)	53 (6.2)	
Median (IQR)	1085 (279.8–7938)	336 (87.5–2925)	0.015
**Residual tumor type**^c^			-
Local	50 (26.3)	-	
Regional	111 (58.4)	-	
Locoregional	29 (15.3)	-	

*Abbreviations*: *T* tumor, *N* lymph node(s), *TNM* tumor-lymph node-metastasis, *EBV* Epstein-Barr virus, *DNA* deoxyribonucleic acid, *IQR* interquartile range, *RT* radiotherapy, *CCRT* concurrent chemoradiotherapy, *NACT* neoadjuvant chemotherapy

^a^According to the World Health Organization (WHO) histologic classification (2005)

^b^All patients’ diseases were re-staged according to the 8th edition of the American Joint Committee on Cancer (AJCC)

^c^The definition of residual tumor types, including local residue: residual tumor in the nasopharynx, and(or) extension to the oropharynx, nasal cavity, parapharyngeal space, adjacent soft tissue, and/or infiltration of bony structures at the skull base, cervical vertebra, pterygoid structures, paranasal sinuses, intracranial extension; regional residue: residual tumor in retropharyngeal lymph nodes and/or cervical lymph nodes; locoregional residue: both local and regional residues

^e^Pearson’s chi-squared test or Fisher’s exact test for categorical variables and Mann–Whitney *U* test for non-normally distributed variables were used to analyse patient characteristics between the two groups

**Fig. 2:**
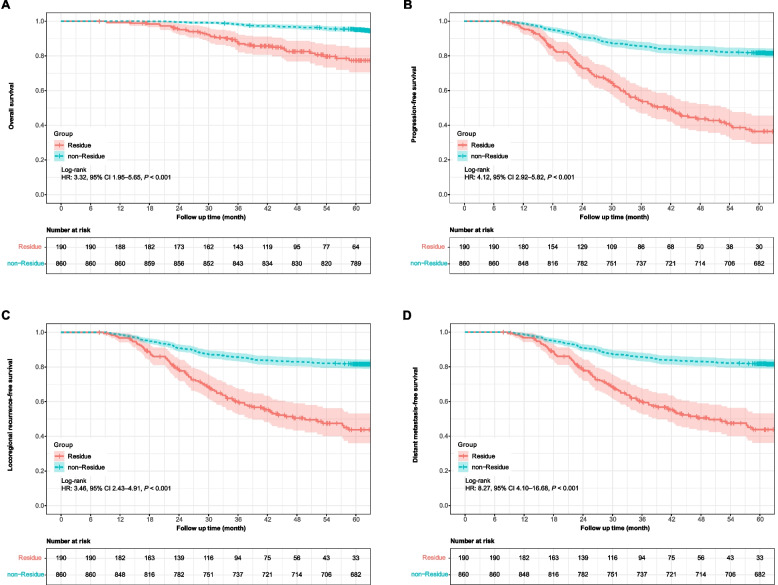
Kaplan–Meier curves for overall survival (**A**), progression free survival (**B**), locoregional recurrence free survival (**C**), and distant metastasis free survival (**D**) in patients with or without residue (*n*=1050)

The estimated 5-year survival rates were lower in patients with residue than in those without it (OS, 77.4% vs. 95.1%; PFS, 36.4% vs. 81.7%; LRRFS, 43.8% vs. 81.7%; DMFS, 78.2% vs. 97.6%; all log-rank *P*<0.001; Fig. 2). No significant differences were observed within the residual tumor types (all log-rank *P*>0.05; sFig. 1). The results of the univariate and multivariate Cox regression analyses with respect to the different endpoints are listed in Table 2. Tumor residue was an independent prognostic factor for OS (hazard ratio (HR) 3.06 [95% confidence interval (CI) 2.00–4.68], *P*<0.001), PFS (HR 4.47 [3.43–5.82], *P*<0.001), LRRFS (HR 3.61 [2.75–4.73], *P*<0.001), and DMFS (HR 8.02 [4.62–13.92], *P*<0.001).

**Table 2:** Univariate and multivariate Cox regression model identified independent prognostic variables

**Endpoint**	**Variables**^a^	**Univariate**		**Multivariate**	
		HR (95% CI)	*P*	HR (95% CI)	*P*
**OS**	Tumor residue	3.54 (2.34–5.35)	<0.001	3.06 (2.00–4.68)	<0.001
	Age ≥ 45 years	2.26 (1.51–3.38)	<0.001	2.27 (1.52–3.40)	<0.001
	Clinical stage	-	<0.001	-	<0.001
	Clinical stage II	Reference	Reference	Reference	Reference
	Clinical stage III	2.94 (1.05–8.20)	0.040	2.62 (0.94–7.32)	0.067
	Clinical stage IVA	6.89 (2.50–19.02)	<0.001	4.98 (1.78–13.90)	0.002
	RT dose 70.00–74.00 Gy	2.25 (1.44–3.51)	<0.001	2.18 (1.39–3.42)	0.001
	Postradiotherapy EBV DNA level copies/ml	-	<0.001	-	-
	0	Reference	Reference	-	-
	1–499	2.01 (0.97–4.17)	0.060	-	-
	≥500	2.63 (1.50–4.61)	0.001	-	-
**PFS**	Tumor residue	4.39 (3.42–5.64)	<0.001	4.47 (3.43–5.82)	<0.001
	Age ≥ 45 years	1.28 (1.00–1.62)	0.048	-	-
	Clinical stage	-	<0.001	-	0.007
	Clinical stage II	Reference	Reference	Reference	Reference
	Clinical stage III	1.59 (1.01–2.50)	0.047	1.25 (0.79–1.97)	0.349
	Clinical stage IVA	3.02 (1.92–4.75)	<0.001	1.78 (1.12–2.85)	0.015
	RT dose 70.00–74.00 Gy	2.37 (1.83–3.09)	<0.001	2.73 (2.09–3.57)	<0.001
	Pretreatment EBV DNA level copies/ml	-	0.006	-	0.047
	0	Reference	Reference	Reference	Reference
	1–4999	1.69 (1.21–2.37)	0.002	1.45 (1.03–2.03)	0.032
	≥5000	1.62 (1.16–2.28)	0.005	1.11 (0.78–1.57)	0.562
	Postradiotherapy EBV DNA level copies/ml	-	<0.001	-	-
	0	Reference	Reference	-	-
	1–499	2.52 (1.63–3.87)	<0.001	-	-
	≥500	2.53 (1.73–3.69)	<0.001	-	-
**LRRFS**	Tumor residue	3.61 (2.78–4.68)	<0.001	3.61 (2.75–4.73)	<0.001
	Clinical stage	-	<0.001	-	0.006
	Clinical stage II	Reference	Reference	Reference	Reference
	Clinical stage III	1.82 (1.12–2.96)	0.016	1.55 (0.95–2.52)	0.080
	Clinical stage IVA	3.24 (1.99–5.26)	<0.001	2.07 (1.26–3.40)	0.004
	RT dose 70.00–74.00 Gy	2.51 (1.91–3.29)	<0.001	2.68 (2.04–3.54)	<0.001
	Pretreatment EBV DNA level copies/ml	-	0.019	-	-
	0	Reference	Reference	-	-
	1–4999	1.59 (1.14–2.24)	0.007	-	-
	≥5000	1.52 (1.08–2.15)	0.017	-	-
	Postradiotherapy EBV DNA level copies/ml	-	<0.001	-	-
	0	Reference	Reference	-	-
	1–499	2.43 (1.55–3.81)	<0.001	-	-
	≥500	2.47 (1.67–3.65)	<0.001	-	-
**DMFS**	Tumor residue	9.19 (5.41–15.60)	<0.001	8.02 (4.62–13.92)	<0.001
	Clinical stage	-	<0.001	-	0.046
	Clinical stage II	Reference	Reference	Reference	Reference
	Clinical stage III	0.80 (0.34–1.90)	0.611	0.64 (0.27–1.52)	0.311
	Clinical stage IVA	2.47 (1.09–5.58)	0.029	1.32 (0.57–3.06)	0.518
	Pretreatment EBV DNA level copies/ml	-	0.055	-	-
	0	Reference	Reference	-	-
	1–4999	2.10 (0.94–4.72)	0.072	-	-
	≥5000	2.87 (1.32–6.26)	0.008	-	-
	Postradiotherapy EBV DNA level copies/ml	-	0.003	-	-
	0	Reference	Reference	-	-
	1–499	2.73 (1.16–6.40)	0.021	-	-
	≥500	2.90 (1.40–5.98)	0.004	-	-

*Abbreviations*: *RT* radiotherapy, *EBV* Epstein–Barr virus, *DNA* deoxyribonucleic acid, *HR* hazard ratio, *CI* confidence interval, *OS* overall survival, *PFS* progression-free survival, *LRRFS* locoregional relapse-free survival, *DMFS* distant metastasis-free survival

^a^The following variables were included in the Cox proportional hazards model multivariate analysis with forward elimination (LR): age (< 45 vs. ≥ 45 years), sex (male vs. female), clinical stage (III vs. III vs. IVA), pretreatment EBV DNA level (0 vs. 1–4999 vs. ≥5000 copies/ml), RT dose to primary ± metastatic tumor sites (68.00–69.96 vs. 70.00–74.00 Gy), postradiotherapy EBV DNA level (0 vs. 1–499 vs. ≥500 copies/ml), and tumor residue (no vs. yes)

### Pretreatment and postradiotherapy plasma EBV DNA level

To develop and validate a prediction nomogram for tumor residue, the patients were randomly divided into a development cohort (70%) and an internal validation cohort (30%) (sTable 1). In the development cohort, pretreatment EBV DNA was detectable in a higher proportion of patients with more advanced disease, as well as a higher median EBV DNA level was observed (*P*<0.001; sTable 2, sFig. 2A). Similarly, this biomarker was also detectable in a higher proportion of patients with a more advanced disease stage, as well as an elevated median level of postradiotherapy (*P*= 0.007; sTable 3, sFig. 2B). A similar distribution was observed in the validation cohort (pretreatment: *P*<0.001, postradiotherapy: *P*=0.019; sTables 2 and 3).

To select the optimal EBV DNA cut-off for predicting tumor residue, we compared the AUC at different cut-off values of EBV DNA in the development cohort (sTables 4 and 5). We first selected the group with three categories of post-treatment EBV DNA cut-off (0 vs. 1–499 vs. ≥500 copies/ml), which provided the highest AUC of 0.627 (0.567–0.688) (sTable 5). The AUCs for three categories (0 vs. 1–4999 vs. ≥5000 copies/ml) and four categories (0 vs. 1–4999 vs. 5000–19999 vs. ≥20000 copies/ml) were almost identical (0.593 vs. 0.604) (sTable 4). The group with three categories in stratifying pretreatment EBV DNA levels (0 vs. 1–4999 vs. ≥5000 copies/ml) was selected for coherence.

The survival rates between subgroups with different pretreatment and postradiotherapy EBV DNA levels were also studied. Both elevated pretreatment and postradiotherapy EBV DNA levels were significantly associated with inferior 5-year survival rates (sFigs. 3 and 4). Moreover, the postradiotherapy EBV DNA level showed better risk discrimination than the pretreatment level (sFigs. 3 and 4; and Table 2). The levels of pretreatment and postradiotherapy EBV DNA are presented in sTable 6.

### Identification of independent predictors of tumor residue

In univariate logistic regression analyses within the development cohort, tumor (T) category, lymph node (N) category, clinical stage, RT dose to nasopharynx ± metastatic cervical lymph node(s), pretreatment, and postradiotherapy EBV DNA levels were found to be significant factors associated with residue (Table 3). Ultimately, clinical stage, RT dose, and postradiotherapy plasma EBV DNA levels were independent predictors of residue after multivariate logistic regression analysis (Table 3).

**Table 3:** Logistic regression analysis of variables for tumor residue in development cohort

Variables	Univariate analysis		Multivariable analysis	
	OR (95% CI)	*P*	OR (95% CI)	*P*
Female	0.78 (0.48–1.25)	0.298	-	-
Age ≥45 years	1.30 (0.88–1.92)	0.186	-	-
Histological type III	0.67 (0.14–3.26)	0.619	-	-
T category	-	-	-	-
T3	1.28 (0.77–2.14)	0.340	-	-
T4	2.28 (1.27–4.07)	0.005	-	-
N category	-	-	-	-
N1	1.32 (0.49–3.54)	0.577	-	-
N2	1.46 (0.54–3.91)	0.453	-	-
N3	4.03 (1.48–11.00)	0.006	-	-
Clinical stage	-	-	-	-
Clinical stage III	2.94 (1.14–7.58)	0.026	2.41 (1.07–7.20)	0.045
Clinical stage IVA	7.86 (3.07–20.12)	<0.001	6.44 (2.67–19.23)	<0.001
Pretreatment EBV DNA level copies/ml	-	-	-	-
1–4999	2.23 (1.24–4.02)	0.007	-	-
≥5000	2.83 (1.59–5.04)	<0.001	-	-
RT dose to nasopharynx ± metastatic cervical lymph node(s) 70.00–74.00 Gy	0.66 (0.44–0.97)	0.035	0.52 (0.34–0.81)	0.004
Postradiotherapy EBV DNA level copies/ml	-	-	-	-
1–499	3.76 (1.74–8.12)	0.001	3.25 (1.41–7.16)	0.004
≥500	13.67 (6.76–27.63)	<0.001	11.92 (5.75–25.88)	<0.001

*Abbreviations*: *T* tumor, *N* lymph node(s), *EBV* Epstein–Barr virus, *DNA* deoxyribonucleic acid, *RT* radiotherapy, *OR* odds ratio

### Prediction nomogram for tumor residue

**Fig. 3:**
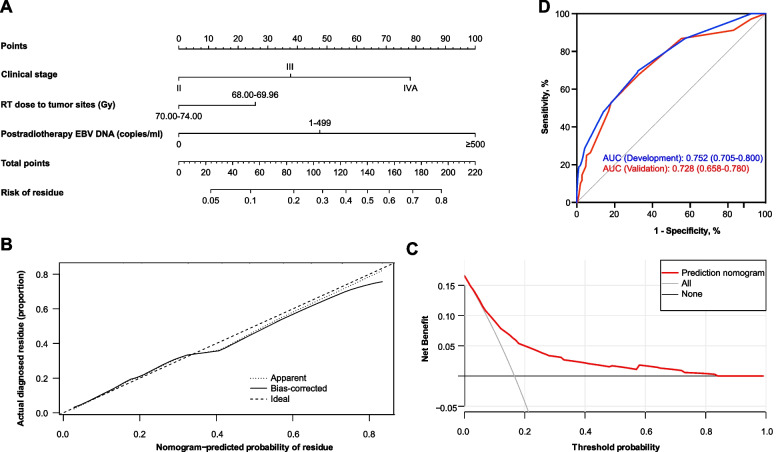
The prediction nomogram of residue (**A**), calibration plot (**B**) and decision curve analysis (**C**) for the prediction nomogram in the development cohort. The receiver operator characteristic curves (**D**) for the prediction nomogram in the development cohort (*n*=736) and validation cohort (*n*=314)

A prediction nomogram for tumor residues, based on these three independent predictors, was established. By summing the weight of every element and corresponding it on a point scale, the probability of residue was estimated for individual patients (Fig. 3A). The nomogram yielded an AUC of 0.752 (95% CI 0.705–0.800) in the development cohort (Fig. 3B), which performed better than the other models (all *P*<0.001) (sFig. 5). The calibration curve of the nomogram showed good agreement between the predicted probability of residue and observed outcomes (Fig. 3C). The DCA plot indicated that the model adds more net benefit when the high-risk threshold for a patient is within a range of 0.07–0.83 (Fig. 3D).

### Validation of the prediction nomogram

**Fig. 4:**
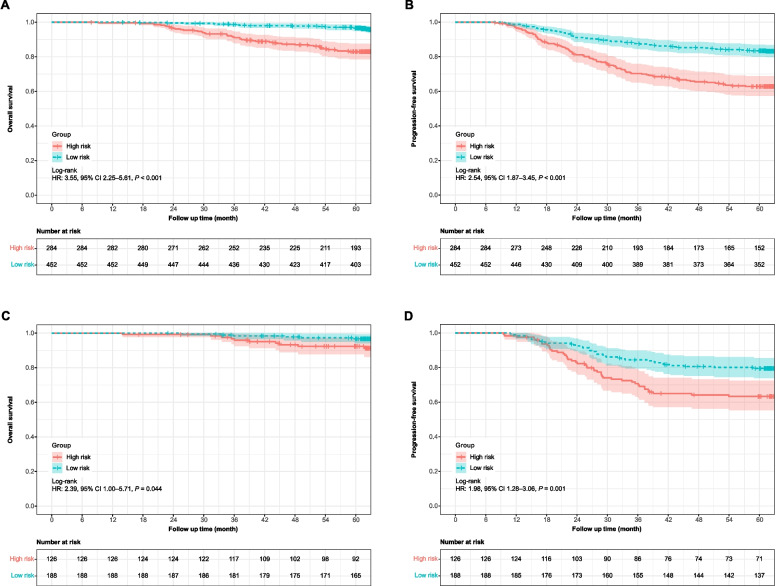
Kaplan–Meier curves for overall survival (**A**) and progression free survival (**B**) in the development cohort (*n*=736) and validation cohort (**C**, **D**) (*n*=314)

The nomogram was further tested in an internal cohort (*n*=314) with an AUC of 0.728 (95% CI, 0.658–0.780) (Fig. 3B). We then calculated the risk points for the individuals using the nomogram. By leveraging the Youden method on the ROC curve, the optimal cut-off for best splitting patients (residue vs. non-residue) was determined to be 69 (sensitivity: 69.67% [95% CI 61.02–77.13], specificity: 67.59% [63.79–71.17]) in the development cohort, and the same value was applied to the validation cohort. The patients were then divided into two groups (low-risk, <69; high-risk, ≥69). High-risk patients in the development and validation cohorts had significantly worse survival outcomes (Fig. 4, sFigs. 6 and 7). In addition, significantly inferior survival outcomes were observed in the high-risk subgroup among patients with residue (sFig. 8 and 9).

## Discussion

Despite the advancement of IMRT, 3–13% of patients with NPC experienced tumor residue 3–6 months after radical RT [10, 11]. Over the past two decades, considerable efforts have been made to investigate the prognostic value of [31–34], compare the capability to diagnose and differentiate residues among available medical procedures [35, 36], and develop predictive models for prognostic stratification and risk adjustment [32] in this field. Unfortunately, little is known about the forecasting tumor residue for the preventive effects at the end of RT. In a large retrospective cohort, we developed and validated a nomogram, integrating clinical stage, postradiotherapy plasma EBV DNA, and RT dose, to estimate the probability of tumor residue after 3–6 months in stage II–IVA NPC patients treated with curative IMRT. The comprehensive nomogram showed better discrimination than clinical stage or postradiotherapy EBV DNA level alone. To the best of our knowledge, this study is the first to investigate the role of postradiotherapy status in predicting residue after 3–6 months.

The tumor-derived EBV DNA load in the plasma represents a microscopic disease in NPC [3]. A detectable or higher level of this pretreatment liquid biomarker is associated with worse outcomes and inferior survival [37]. While it can be eradicated during treatment in most patients, 9.2–28.8% of patients have microscopic tumor residue in the circulation after treatment [18, 38, 39]. Chan et al. conducted several prospective studies to investigate their prognostic significance. In 170 NPC patients receiving a uniform RT protocol, 28.8% of patients had detectable posttreatment EBV DNA (at 6–8 weeks after RT) and exhibited inferior PFS (HR 11.9, 95% CI 5.53–25.43) and OS (HR 8.6, 95% CI 3.69–19.97) compared to patients with higher pretreatment EBV DNA [18]. Similarly, a better risk discrimination concerning different endpoints in the postradiotherapy EBV DNA level than pretreatment level was observed in our study. In a large prospective plasma EBV DNA screening study for identification of high-risk NPC patients for adjuvant chemotherapy, the positive relationship between detectable or higher levels of postradiotherapy EBV DNA (within 120 days) and disease failures was highlighted. Unfortunately, patients with detectable postradiotherapy liquid biomarker levels did not benefit from adjuvant chemotherapy [38]. Therefore, detectable postradiotherapy EBV DNA levels are not a determinant factor in identifying high-risk patients. In their post-hoc analysis, a combination of postradiotherapy EBV DNA and clinical stage improved risk stratification for NPC using recursive partitioning analysis compared to either the clinical stage or postradiotherapy EBV DNA alone (concordance-index (C-index): 0.712 vs. 0.604 vs. 0.675) [39]. The aforementioned series of investigations implied the potential of a combination of clinical stage and postradiotherapy EBV DNA level in predicting tumor residue. In our study, plasma EBV DNA was detectable in 10.6% of patients after RT, consistent with a previous study [39]. In Chan’s study, a postradiotherapy EBV DNA level yielded a C-index of 0.675 in predicting 5-year OS [39]. Tumor residue is an early failure pattern that can be plausibly predicted by postradiotherapy EBV DNA which has been related to minimal residual disease at the end of RT. In our study, the postradiotherapy EBV DNA level alone achieved a higher AUC in predicting residue compared to the pretreatment EBV DNA level (0.627 vs. 0.593). The performance in predicting tumor residue was improved by a combination of clinical stage and posttreatment liquid biomarkers (AUC: 0.733), which verified the aforementioned hypothesis.

In this study, 69.96 Gy was employed as a cut-off value for sectionalisation for the RT dose. The reason for choosing this prescribed dose was that it was uniformly recommended to all NPC patients for its good balance between the tumor-killing effect and normal tissue tolerance according to the National Comprehensive Cancer Network. Notably, the RT dose to the primary tumor site and involved lymph nodes was significantly associated with tumor residue in our model. This result demonstrated that the tumor residue is associated with tumor radiation insensitivity or an insufficient dose. This is likely a reflection of the underlying biological heterogeneity of patients with NPC. We reasoned that a uniformly prescribed dose may be insufficient for patients with potential radiation resistance. Several studies have shown that boost RT dose for residual lesions can improve tumor control and survival rates [40, 41]. Fei et al. conducted a study on 398 NPC patients with T4 stage disease who had local residue after radical IMRT (70 Gy). In their study, 114 patients received boost dose of 4–6.75 Gy in local residual lesions (2–3 fractions, 2–2.25 Gy per fraction, 1 fraction per day, 5 fractions per week). After follow-up, the boost group exhibited better 3-year OS (86.6% vs. 71.3%, *P*=0.008), PFS (79.0% vs. 69.1%, *P*=0.019), and LRRFS (93.4% vs. 82.4%, *P*=0.012) than the non-boost group [14]. In our study, patients receiving a dose under 70.00 Gy were more likely to have residue, which implies that a dose boost may reverse the outcome. Meanwhile, others argue that the boost dose should not be delivered indiscreetly if the delivered dose for the gross tumor volume is sufficient. In a retrospective study by Han et al., the tumor residual rate was 6.1% (12/196) three months after IMRT. All 12 residual lesions resolved completely 4–9 months after RT [42]. In the era of IMRT, the blind administration of additional RT to the residual tumor seems unwise. Thus, the challenge lies in patient selection to maximise the magnitude of the benefit. Although the prediction nomogram built in our study cannot predict residual tumor pretherapy, we provided a compromise at the end of IMRT. After receiving a uniform standard dose, timely salvage treatment is necessary for patients at risk of tumor residue.

However, this study has some limitations. First, it was a retrospective study. Second, the timing of postradiotherapy plasma EBV DNA levels was not standardised within the studied patients. In most clinical trials of adjuvant therapy for NPC, the therapy is usually planned to start within 4 weeks after the completion of RT. To cover this range, we extended the time of measuring plasma EBV DNA levels to 4 weeks after RT. Third, our prediction nomogram was built and validated at a single center. Further external validation will help to attain high-level evidence of clinical feasibility. Also, prospective clinical studies with large cohorts are warranted to investigate strategies for tumor residue prevention.

## Conclusions

In summary, we developed a nomogram integrating clinical stage, postradiotherapy plasma EBV DNA level, and RT dose for the primary tumor and metastatic lymph nodes for predicting whether tumor residue or not after 3–6 months in patients with stage II–IVA NPC disease after completion of IMRT. The proposed nomogram proved to be a reliable tool for estimating residue risk and appears to provide an opportunity for timely improvement or consolidation of efficacy.

## Supplementary Information

**Additional file 1: sTable 1.** Clinical characteristics of patients in the development and validation cohorts. **sTable 2.** Distribution of pretreatment plasma EBV DNA by TNM stage (AJCC 8^th^ edition). **sTable 3.** Distribution of postradiotherapy plasma EBV DNA by TNM stage (AJCC 8^th^ edition)**.** **sTable 4.** Comparison of AUC and OR of residue at different level of pretreatment EBV DNA in the development cohort (*n*=736). **sTable 5.** Comparison of AUC and OR of residue at different level of postradiotherapy EBV DNA in the development cohort (*n*=736). **sTable 6.** Pretreatment and postradiotherapy levels of EBV DNA in all patients (*n*=1050). **sFigure 1.** Kaplan-Meier curves for overall survival (A), progression-free survival (B), locoregional recurrence-free survival (C) and distant metastasis-free survival (D) in patients stratified by residual tumor type (*n*=190). **sFigure 2.** Pretreatment (A) and postradiotherapy (B) plasma EBV DNA levels by TNM stage (AJCC 8^th^ edition) in the development cohort (*n*=736). **sFigure 3.** Kaplan-Meier curves for overall survival (A), progression-free survival (B), locoregional recurrence-free survival (C) and distant metastasis-free survival (D) in all patients stratified by pretreatment plasma EBV DNA level (*n*=1050). **sFigure 4.** Kaplan-Meier curves for overall survival (A), progression-free survival (B), locoregional recurrence-free survival (C) and distant metastasis-free survival (D) in all patients stratified by postradiotherapy plasma EBV DNA level (*n*=1050). **sFigure 5.** Comparison of six models using area under the receiver operating characteristic curve calculated for the development cohort (*n*=736). **sFigure 6.** Kaplan-Meier curves for locoregional recurrence-free survival (A) and distant metastasis-free survival (B) in development cohort stratified by predicted risk in tumor residue (*n*=736). **sFigure 7.** Kaplan-Meier curves for locoregional recurrence-free survival (A) and distant metastasis-free survival (B) in validation cohort stratified by predicted risk in tumor residue (*n*=314). **sFigure 8.** Kaplan-Meier curves for overall survival (A), progression free-survival (B), locoregional recurrence-free survival (C) and distant metastasis-free survival (D) in patients with residue from development cohort stratified by predicted risk in tumor residue (*n*=122). **sFigure 9.** Kaplan-Meier curves for overall survival (A), progression free-survival (B), locoregional recurrence-free survival (C) and distant metastasis-free survival (D) in patients with residue from validation cohort stratified by predicted risk in tumor residue (*n*=68).

## Data Availability

The authenticity of this article has been validated by uploading the key raw data onto the Research Data Deposit public platform (www.researchdata.org.cn) with approval RDD number (RDDA2022547695). All data will be shared upon request to the corresponding author.

## Abbreviations

EBV: Epstein–Barr virus
DNA: Deoxyribonucleic acid
RT: Radiotherapy
NPC: Nasopharyngeal carcinoma
IMRT: Intensity-modulated radiation therapy
AUC: Area under the curve
LA-NPC: Locoregionally advanced nasopharyngeal carcinoma
18F-FDG: 18F-fluorodeoxyglucose
OS: Overall survival
PFS: Progression-free survival
LRRFS: Locoregional relapse-free survival
DMFS: Distant metastasis-free survival
ROC: Receiver operating characteristic
DCA: Decision curve analysis
HR: Hazard ratio
CI: Confidence interval
C-index: Concordance-index
