# Supplementary material for: Tumor residue in patients with stage II–IVA nasopharyngeal carcinoma who received intensity-modulated radiation therapy: development and validation of a prediction nomogram integrating postradiotherapy plasma Epstein–Barr virus deoxyribonucleic acid, clinical stage, and radiotherapy dose
Source: BMC Cancer. 2023 May 6;23:410. doi: 10.1186/s12885-023-10827-0 (PMC10164328; doi:10.1186/s12885-023-10827-0)
Supplement: Supplementary file 1 — Additional file 1: sTable 1. Clinical characteristics of patients in the development and validation cohorts. sTable 2. Distribution of pretreatment plasma EBV DNA by TNM stage (AJCC 8th edition). sTable 3. Distribution of postradiotherapy plasma EBV DNA by TNM stage (AJCC 8th edition). sTable 4. Comparison of AUC and OR of residue at different level of pretreatment EBV DNA in the development cohort (n=736). sTable 5. Comparison of AUC and OR of residue at different level of postradiotherapy EBV DNA in the development cohort (n=736). sTable 6. Pretreatment and postradiotherapy levels of EBV DNA in all patients (n=1050). sFigure 1. Kaplan-Meier curves for overall survival (A), progression-free survival (B), locoregional recurrence-free survival (C) and distant metastasis-free survival (D) in patients stratified by residual tumor type (n=190). sFigure 2. Pretreatment (A) and postradiotherapy (B) plasma EBV DNA levels by TNM stage (AJCC 8th edition) in the development cohort (n=736). sFigure 3. Kaplan-Meier curves for overall survival (A), progression-free survival (B), locoregional recurrence-free survival (C) and distant metastasis-free survival (D) in all patients stratified by pretreatment plasma EBV DNA level (n=1050). sFigure 4. Kaplan-Meier curves for overall survival (A), progression-free survival (B), locoregional recurrence-free survival (C) and distant metastasis-free survival (D) in all patients stratified by postradiotherapy plasma EBV DNA level (n=1050). sFigure 5. Comparison of six models using area under the receiver operating characteristic curve calculated for the development cohort (n=736). sFigure 6. Kaplan-Meier curves for locoregional recurrence-free survival (A) and distant metastasis-free survival (B) in development cohort stratified by predicted risk in tumor residue (n=736). sFigure 7. Kaplan-Meier curves for locoregional recurrence-free survival (A) and distant metastasis-free survival (B) in validation cohort stratified by predicted risk in t [file 12885_2023_10827_MOESM1_ESM.pdf]

Supplementary Material

Tumor residue in patients with stage II–IVA nasopharyngeal carcinoma who received intensity-modulated radiation therapy: development and validation of a prediction nomogram integrating postradiotherapy plasma Epstein–Barr virus deoxyribonucleic acid, clinical stage, and radiotherapy dose

Supplementary Tables

sTable 1. Clinical characteristics of patients in the development and validation cohorts

sTable 2. Distribution of pretreatment plasma EBV DNA by TNM stage (AJCC 8th edition)

sTable 3. Distribution of postradiotherapy plasma EBV DNA by TNM stage (AJCC 8th edition)

sTable 4. Comparison of AUC and OR of residue at different level of pretreatment EBV DNA in the development cohort (n=736)

sTable 5. Comparison of AUC and OR of residue at different level of postradiotherapy EBV DNA in the development cohort (n=736)

sTable 6. Pretreatment and postradiotherapy levels of EBV DNA in all patients (n=1050)

Supplementary Figures

sFigure 1. Kaplan-Meier curves for overall survival (A), progression-free survival (B), locoregional recurrence-free survival (C) and distant metastasis-free survival (D) in patients stratified by residual tumor type (n=190)

sFigure 2. Pretreatment (A) and postradiotherapy (B) plasma EBV DNA levels by TNM stage (AJCC 8th edition) in the development cohort (n=736)

sFigure 3. Kaplan-Meier curves for overall survival (A), progression-free survival (B), locoregional recurrence-free survival (C) and distant metastasis-free survival (D) in all patients stratified by pretreatment plasma EBV DNA level (n=1050)

sFigure 4. Kaplan-Meier curves for overall survival (A), progression-free survival (B), locoregional recurrence-free survival (C) and distant metastasis-free survival (D) in all patients stratified by postradiotherapy plasma EBV DNA level (n=1050)

sFigure 5. Comparison of six models using area under the receiver operating characteristic curve calculated for the development cohort (n=736)

sFigure 6. Kaplan-Meier curves for locoregional recurrence-free survival (A) and distant metastasis-free survival (B) in development cohort stratified by predicted risk in tumor residue (n=736)

sFigure 7. Kaplan-Meier curves for locoregional recurrence-free survival (A) and distant metastasis-free survival (B) in validation cohort stratified by predicted risk in tumor residue (n=314)

sFigure 8. Kaplan-Meier curves for overall survival (A), progression free-survival (B), locoregional recurrence-free survival (C) and distant metastasis-free survival (D) in patients with residue from development cohort stratified by predicted risk in tumor residue (n=122)

sFigure 9. Kaplan-Meier curves for overall survival (A), progression free-survival (B), locoregional recurrence-free survival (C) and distant metastasis-free survival (D) in patients with residue from validation cohort stratified by predicted risk in tumor residue (n=68)

sTable 1. Clinical characteristics of patients in the development and validation cohorts

**Characteristics**	**Development cohort** No. (%)	**Validation cohort** No. (%)	***P***[e]
**Total**	*n*=736 (70.1)	*n*=314 (29.9)	
**Sex**			0.713
Male	558 (75.8)	234 (74.5)	
Female	178 (24.2)	80 (25.5)	
**Age, years**			0.497
<45	420 (57.1)	187(59.6)	
⩾45	316 (42.9)	127 (40.4)	
**Histological type**[a]			1.000
I&II	8 (1.1)	4 (1.3)	
III	728 (98.9)	310 (98.7)	
**T category**[b]			0.414
T2	183 (24.9)	89 (28.3)	
T3	411 (55.8)	172 (54.8)	
T4	142 (19.3)	53 (16.9)	
**N category**[b]			0.782
N0	48 (6.5)	22 (7.0)	
N1	300 (40.8)	129 (41.1)	
N2	269 (36.5)	106 (33.8)	
N3	119 (16.2)	57 (18.2)	
**AJCC TNM stage**[b]			0.875
II	107 (14.5)	49 (15.6)	
III	381 (51.8)	163 (51.9)	
IVA	248 (33.7)	102 (32.5)	
**Pretreatment EBV DNA, copies/ml**			0.585
0	195 (26.5)	80 (25.5)	
1-4999	273 (37.1)	127 (40.4)	
⩾5000	268 (36.4)	107 (34.1)	
No of patients (%) with detectable pretreatment EBV DNA (>0), copies/ml	541 (73.5)	234 (74.5)	
Median (IQR)	4830 (1280-21150)	4135 (1098-18050)	0.569
**Treatment modality**			0.484
RT	66 (9.0)	25 (8.0)	
CCRT	320 (43.5)	149 (47.5)	
NACT+CCRT	350 (47.6)	140 (44.6)	
**RT dose to nasopharynx ± metastatic cervical lymph node(s), Gy**			0.916
68.00-69.96	352 (47.8)	152 (48.4)	
70.00-74.00	384 (52.2)	162 (51.6)	
Median (IQR)	70.00 (69.90-70.06)	70.00 (69.90-70.06)	0.810
**Postradiotherapy EBV DNA, copies/ml**			0.231
0	666 (90.5)	273 (86.9)	

1-499	31 (4.2)	18 (5.7)	
⩾500	39 (5.3)	23 (7.3)	
No of patients (%) with detectable postradiotherapy EBV DNA (>0), copies/ml	70 (9.5)	41 (13.1)	
Median (IQR)	680 (113.8-3385)	950 (142.5-7710)	0.442
**Residual tumor type**[c]			0.103
Local	30 (4.0)	20 (6.2)	
Regional	70 (9.2)	41 (12.7)	
Locoregional	22 (2.9)	7 (2.2)	

Abbreviations: T, tumor; N, lymph node(s); TNM, tumor-lymph node-metastasis; EBV, Epstein-Barr virus; DNA, deoxyribonucleic acid; IQR, interquartile range; RT, radiotherapy; CCRT, concurrent chemoradiotherapy; NACT, neoadjuvant chemotherapy.

[a]According to the World Health Organization (WHO) histologic classification (2005)

[b]All patients' diseases were re-staged according to the 8th edition of the American Joint Committee on Cancer (AJCC)

[c]The definition of residual tumor types, including local residue: residual tumor in the nasopharynx, and(or) extension to the oropharynx, nasal cavity, parapharyngeal space, adjacent soft tissue, and/or infiltration of bony structures at the skull base, cervical vertebra, pterygoid structures, paranasal sinuses, intracranial extension; regional residue: residual tumor in retropharyngeal lymph nodes and/or cervical lymph nodes; locoregional residue: both local and regional residues

[e]Pearson's chi-squared test or Fisher's exact test for categorical variables and Mann–Whitney *U* test for non-normally distributed variables were used to analyse patient characteristics between the two groups

sTable 2. Distribution of pretreatment plasma EBV DNA by TNM stage (AJCC 8th edition)

No of patients (%)	All stage (II-IVA)	AJCC TNM stage II	AJCC TNM stage III	AJCC TNM stage IVA	*P*
**Development cohort**	736 (100.0)	107 (14.5)	381 (51.8)	248 (33.7)	<0.001[a]
Undetectable pretreatment EBV DNA (0 copies/ml)	195 (26.5)	45 (42.1)	103 (27.0)	47 (18.9)	
Detectable pretreatment EBV DNA (>0 copies/ml)	541 (73.5)	62 (57.9)	278 (73.0)	201 (81.1)	
Median (copies/ml)	4830	2925	4450	6575	
Interquartile range	1280-21150	706.5-10185	1128-18125	1963-31300	
**Validation cohort**	314 (100.0)	49 (15.6)	163(51.9)	102 (32.5)	<0.001[a]
Undetectable pretreatment EBV DNA (0 copies/ml)	80 (25.5)	22 (44.9)	44 (27.0)	14 (13.7)	
Detectable pretreatment EBV DNA (>0 copies/ml)	234 (74.5)	27 (55.1)	119 (63.0)	88 (86.3)	
Median (copies/ml)	4135	2680	3010	12900	
Interquartile range	1098-18050	498-15000	690-12200	2128-36850	

Abbreviations: EBV, Epstein-Barr virus; DNA, deoxyribonucleic acid; AJCC, American Joint Committee on Cancer (8th Edition); TNM, tumor-lymph node-metastasis

[a]Correlation of pretreatment EBV DNA (Undetectable vs. Detectable) and AJCC TNM Stage (II, III, IVA) was by Chi-squared test

sTable 3. Distribution of postradiotherapy plasma EBV DNA by TNM stage (AJCC 8th edition)

No of patients (%)	All stage (II-IVA)	AJCC TNM stage II	AJCC TNM stage III	AJCC TNM stage IVA	*P*
**Development cohort**	736 (100.0)	107 (14.5)	381 (51.8)	248 (33.7)	0.007[a]
Undetectable postradiotherapy EBV DNA (0 copies/ml)	666 (90.5)	103 (96.3)	349 (91.6)	214 (86.3)	
Detectable postradiotherapy EBV DNA (>0 copies/ml)	70 (9.5)	4 (3.7)	32 (8.4)	34 (13.7)	
Median (copies/ml)	680	152.3	578.1	970	
Interquartile range	113.8-3385	37.6-912.8	92-3095	135.8-6028	
**Validation cohort**	314 (100.0)	49 (15.6)	163(51.9)	102 (32.5)	0.019[a]
Undetectable postradiotherapy EBV DNA (0 copies/ml)	273 (86.9)	48 (98.0)	140 (85.9)	85 (83.3)	
Detectable postradiotherapy EBV DNA (>0 copies/ml)	41 (13.1)	1 (2.0)	23 (14.1)	17 (16.7)	
Median (copies/ml)	950	1750	488	1030	
Interquartile range	142.5-7710	-	136-10400	196-7585	

Abbreviations: EBV, Epstein-Barr virus; DNA, deoxyribonucleic acid; AJCC, American Joint Committee on Cancer (8th Edition); TNM, tumor-lymph node-metastasis

[a]Correlation of postradiotherapy EBV DNA (Undetectable vs. Detectable) and AJCC TNM Stage (II, III, IVA) was by Chi-squared test.

sTable 4. Comparison of AUC and OR of residue at different level of pretreatment EBV DNA in the development cohort (n=736)

**Level of pretreatment EBV DNA cut-off (copies/ml)**	**AUC (95% CI)**	**OR (95% CI)**	***P*[a]**
EBV DNA: 2 groups (Undetectable vs. Detectable)	0.575 (0.523-0.627)	2.52 (1.47-4.33)	0.001
EBV DNA: 2 groups (0-4999 vs. ≥5000)	0.562 (0.505-0.618)	1.67 (1.13-2.48)	0.010
EBV DNA: 2 groups (0-19999 vs. ≥20000)	0.561 (0.503-0.619)	2.01 (1.29-3.12)	0.002
EBV DNA: 3 groups (0 vs. 1-4999 vs. ≥5000)	0.593 (0.541-0.646)	1.00	-
		2.23 (1.24-4.02)	0.007
		2.83 (1.59-5.04)	<0.001
EBV DNA: 3 groups (0-4999 vs. 5000-19999 vs. ≥20000)	0.572 (0.515-0.630)	1.00	-
		1.24 (0.72-2.12)	0.432
		2.11 (1.33-3.34)	0.002
EBV DNA: 4 groups (0 vs. 1-4999 vs. 5000-19999 vs. ≥20000)	0.604 (0.550-0.657)	1.00	-
		2.23 (1.24-4.02)	0.007
		2.09 (1.06-4.15)	0.034
		3.56 (1.90-6.64)	<0.001

Abbreviations: EBV, Epstein-Barr virus; DNA, deoxyribonucleic acid; AUC, area under curve; OR, odds ratio; CI, confidence interval

[a]Logistic regression model was used to calculate the odds ratio, corresponding 95% CI and *P* value

sTable 5. Comparison of AUC and OR of residue at different level of postradiotherapy EBV DNA in the development cohort (n=736)

**Level of postradiotherapy EBV DNA cut-off (copies/ml)**	**AUC (95% CI)**	**OR (95% CI)**	***P*[a]**
EBV DNA: 2 groups (Undetectable vs. Detectable)	0.625 (0.564-0.685)	7.66 (4.55-12.91)	<0.001
EBV DNA: 2 groups (0-499 vs. ≥500)	0.596 (0.535-0.656)	12.52 (6.22-25.21)	<0.001
EBV DNA: 2 groups (0-1999 vs. ≥2000)	0.556 (0.496-0.615)	12.16 (4.84-30.52)	<0.001
EBV DNA: 3 groups (0 vs. 1-499 vs. ≥500)	0.627 (0.567-0.688)	1.00	-
		3.76 (1.74-8.12)	0.001
		13.67 (6.76-27.63)	<0.001
EBV DNA: 3 groups (0-499 vs. 500-1999 vs. ≥2000)	0.596 (0.536-0.656)	1.00	-
		11.48 (4.15-31.76)	<0.001
		13.42 (5.33-33.75)	<0.001
EBV DNA: 4 groups (0 vs. 1-499 vs. 500-1999 vs. ≥2000)	0.604 (0.550-0.657)	1.00	-
		3.76 (1.74-8.12)	0.001
		12.53 (4.52-34.77)	<0.001
		14.65 (5.80-36.96)	<0.001

Abbreviations: EBV, Epstein-Barr virus; DNA, deoxyribonucleic acid; AUC, area under curve; OR, odds ratio; CI, confidence interval

[a]Logistic regression model was used to calculate the odds ratio, corresponding 95% CI and *P* value

sTable 6. Pretreatment and postradiotherapy levels of EBV DNA in all patients (n=1050)

	**Postradiotherapy EBV DNA, copies/ml**			
**Pretreatment EBV DNA, copies/ml**	0	1-499	⩾500	Total (%)
0	262 (24.9)	8 (0.8)	5 (0.5)	275 (26.2)
1-4999	360 (34.3)	23 (2.2)	17 (1.6)	400 (38.1)
⩾5000	317 (30.2)	18 (1.7)	40 (3.8)	375 (35.7)
Total	939 (89.4)	49 (4.7)	62 (5.9)	1050 (100.0)

Abbreviations: EBV, Epstein-Barr virus; DNA, deoxyribonucleic acid

sFigure 1. Kaplan-Meier curves for overall survival (A), progression-free survival (B), locoregional recurrence-free survival (C) and distant metastasis-free survival (D) in patients stratified by residual tumor type (n=190)

A.

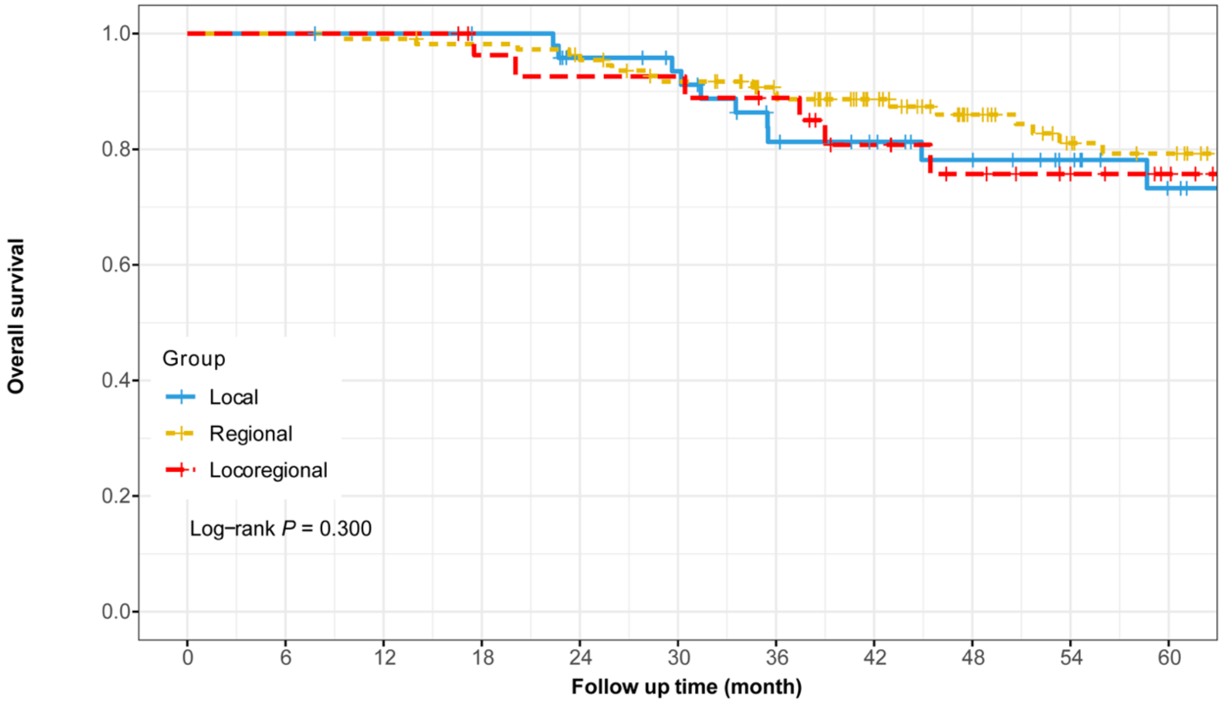

Number at risk

	0	6	12	18	24	30	36	42	48	54	60
Local	50	50	49	48	43	40	32	29	24	20	14
Regional	111	111	110	108	105	97	88	72	57	47	43
Locoregional	29	29	29	26	25	25	23	18	14	10	7

B.

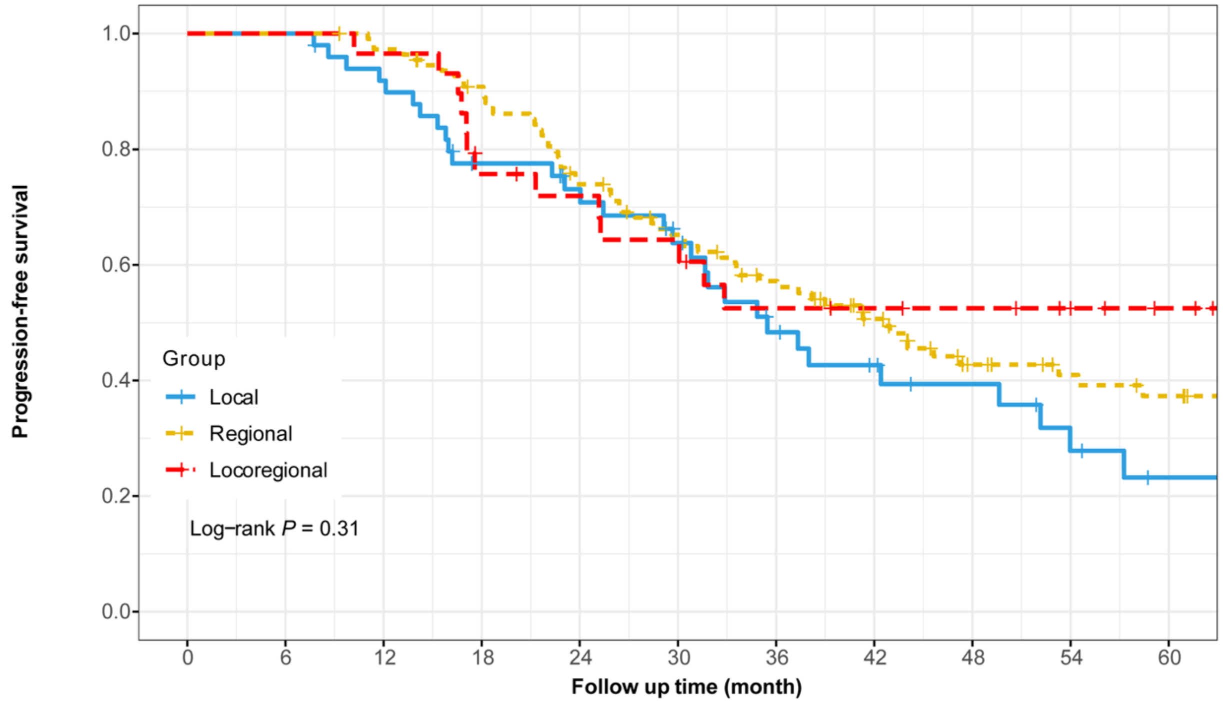

Number at risk

	0	6	12	18	24	30	36	42	48	54	60
Local	50	50	45	36	32	26	18	14	11	7	4
Regional	111	111	107	97	78	66	55	42	28	23	20
Locoregional	29	29	28	21	19	17	13	12	11	8	6

C.

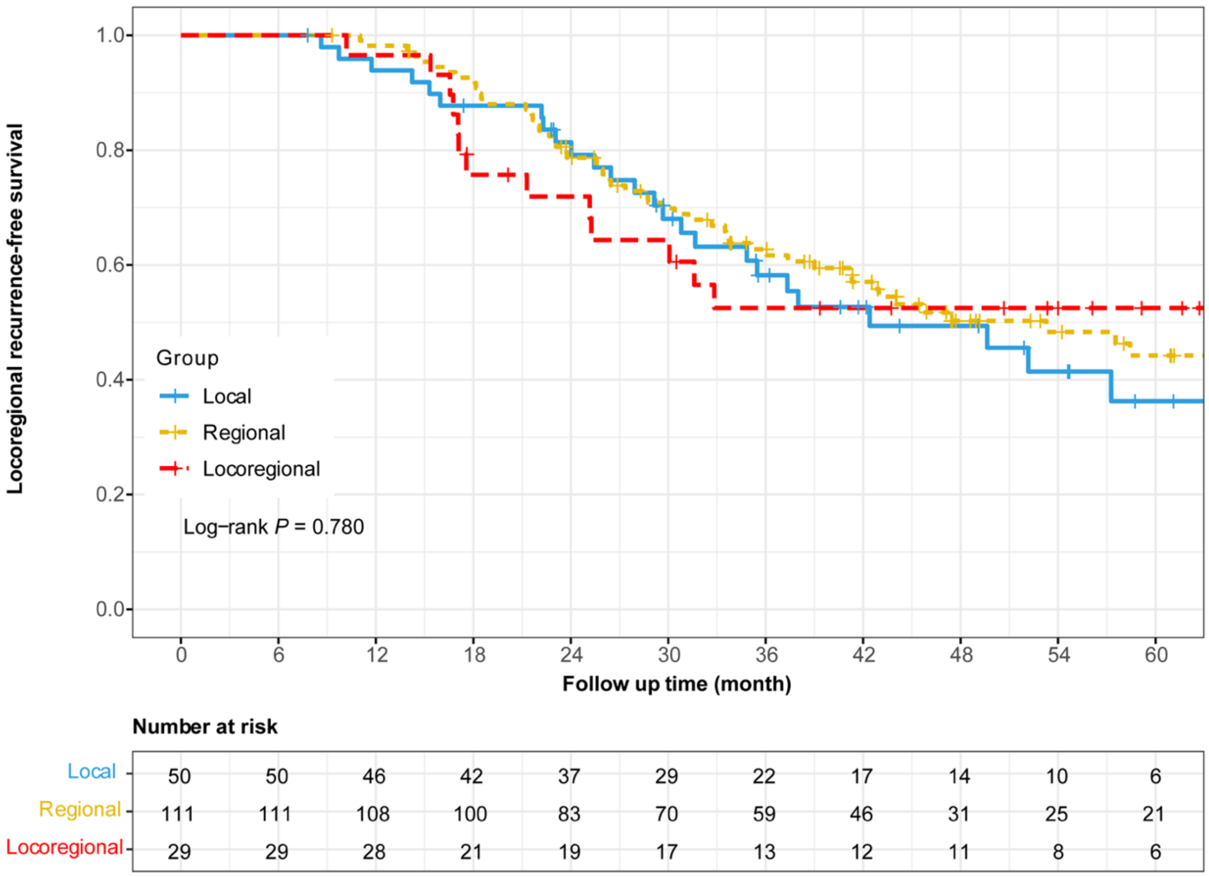
Locoregional recurrence-free survival
1.0
0.8
0.6
0.4
0.2
0.0
Group
Local
Regional
Locoregional
Log−rank P = 0.780
0 6 12 18 24 30 36 42 48 54 60
Follow up time (month)
Number at risk
Local 50 50 46 42 37 29 22 17 14 10 6
Regional 111 111 108 100 83 70 59 46 31 25 21
Locoregional 29 29 28 21 19 17 13 12 11 8 6

D.

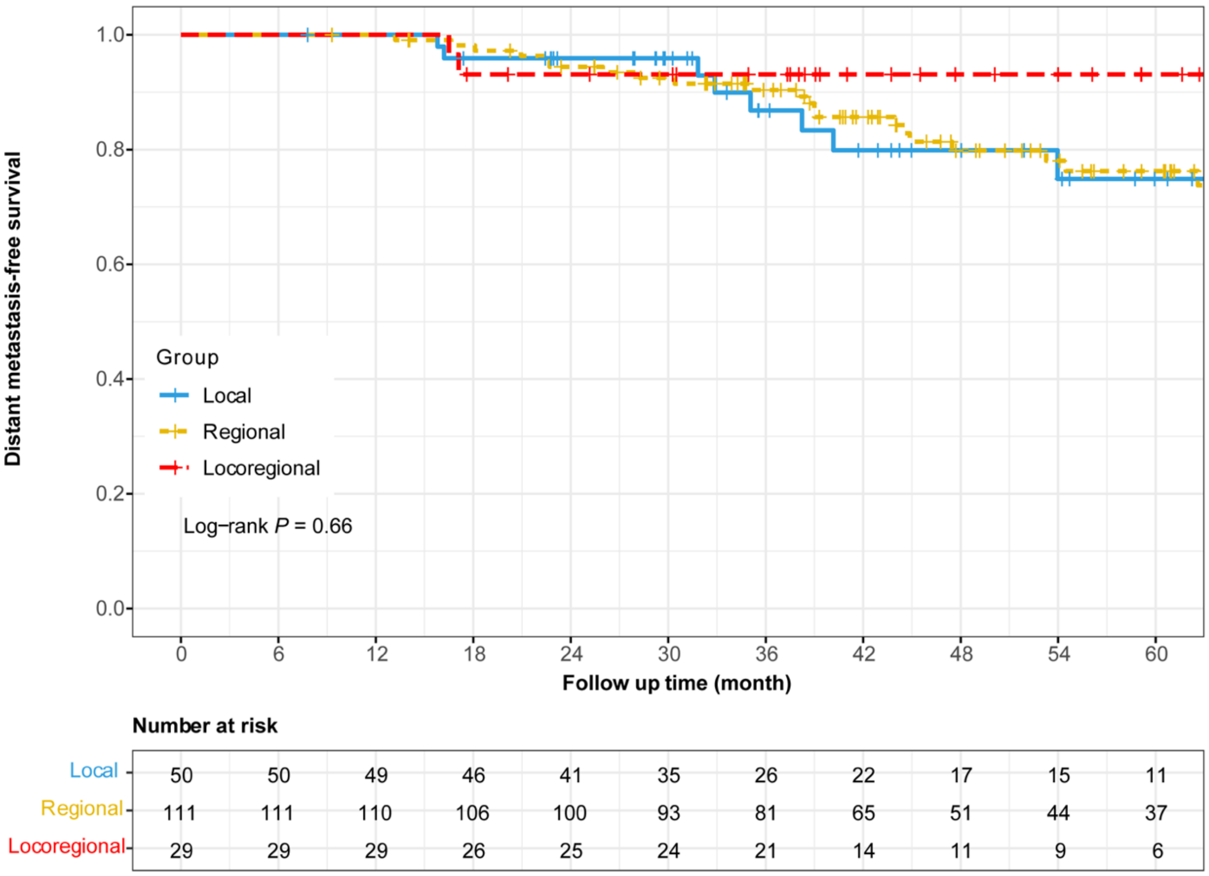
Distant metastasis-free survival
1.0
0.8
0.6
0.4
0.2
0.0
Group
Local
Regional
Locoregional
Log−rank P = 0.66
0 6 12 18 24 30 36 42 48 54 60
Follow up time (month)
Number at risk
Local 50 50 49 46 41 35 26 22 17 15 11
Regional 111 111 110 106 100 93 81 65 51 44 37
Locoregional 29 29 29 26 25 24 21 14 11 9 6

sFigure 2. Pretreatment (A) and postradiotherapy (B) plasma EBV DNA levels by TNM stage (AJCC 8th edition) in the development cohort (n=736)

Abbreviations: EBV, Epstein-Barr virus; DNA, deoxyribonucleic acid

The black line represents the median value. The bottom and top of the dots are the 25th and 75th percentiles (interquartile range).

A.

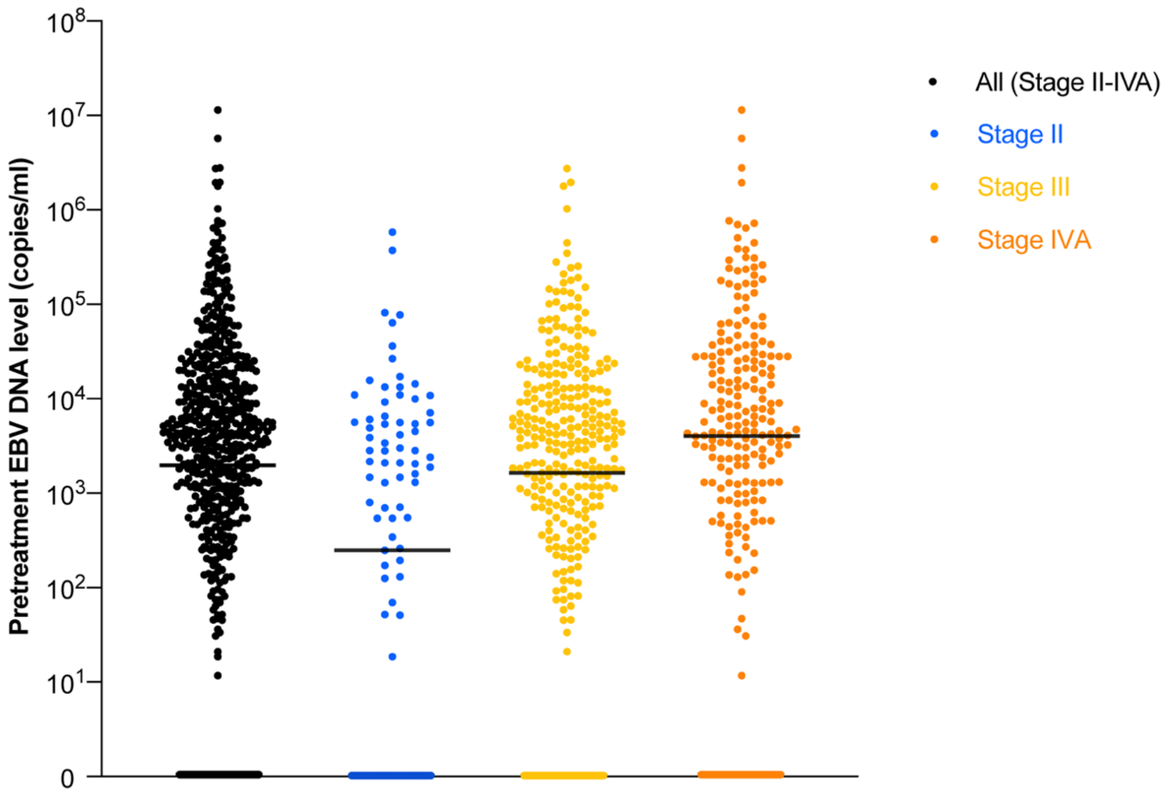

B.

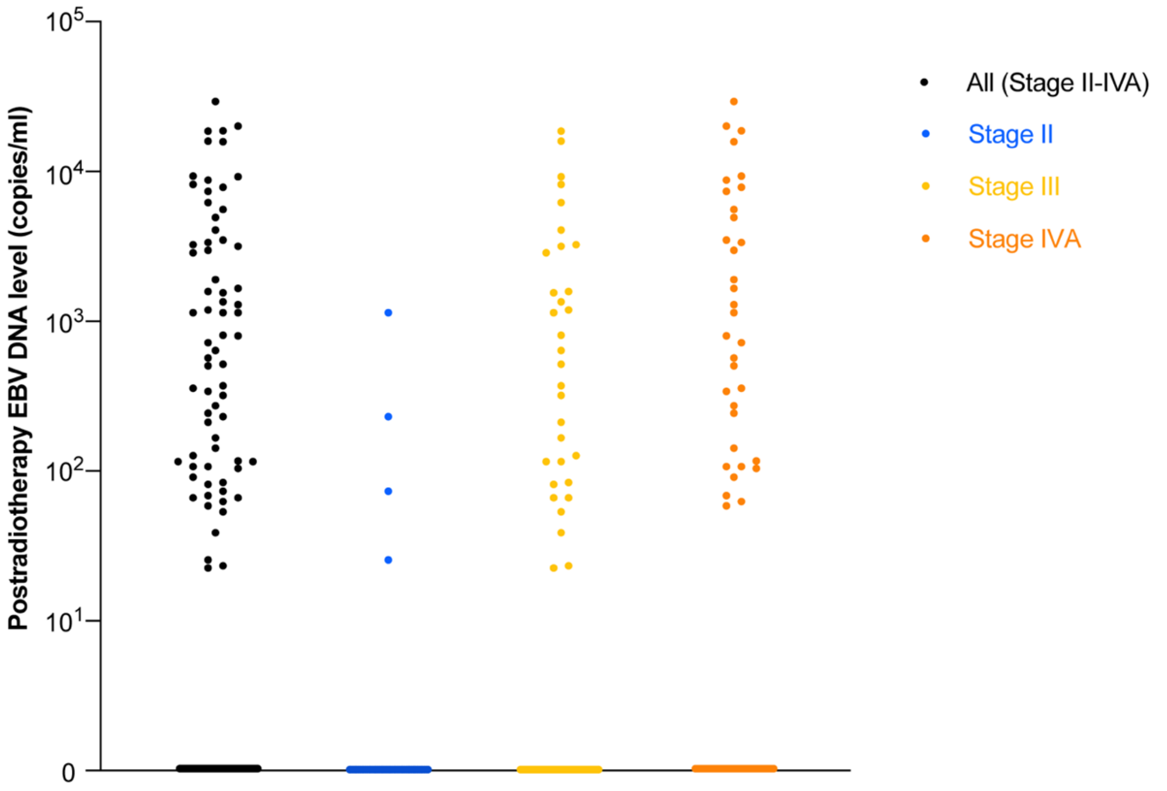

sFigure 3. Kaplan-Meier curves for overall survival (A), progression-free survival (B), locoregional recurrence-free survival (C) and distant metastasis-free survival (D) in all patients stratified by pretreatment plasma EBV DNA level (n=1050)

Abbreviations: EBV, Epstein-Barr virus; DNA, deoxyribonucleic acid

A.

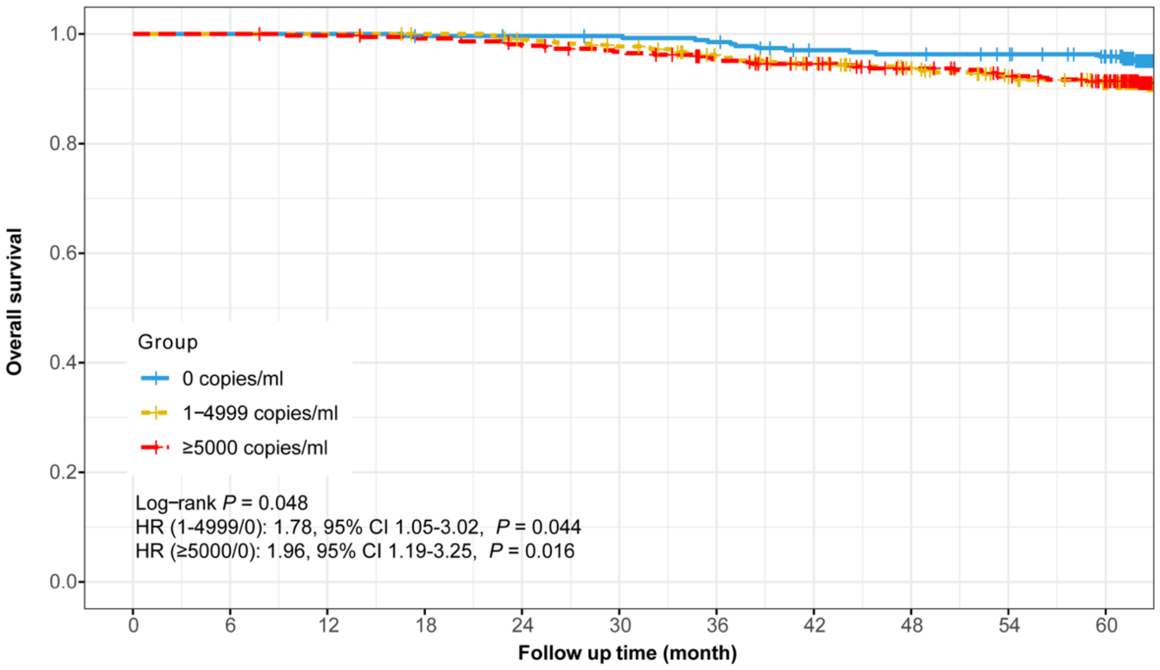

Number at risk

0	275	275	275	273	272	271	268	259	257	254	245
1−4999	400	400	400	398	392	385	371	357	343	328	307
≥5000	375	375	373	370	365	358	347	337	325	315	301

B.

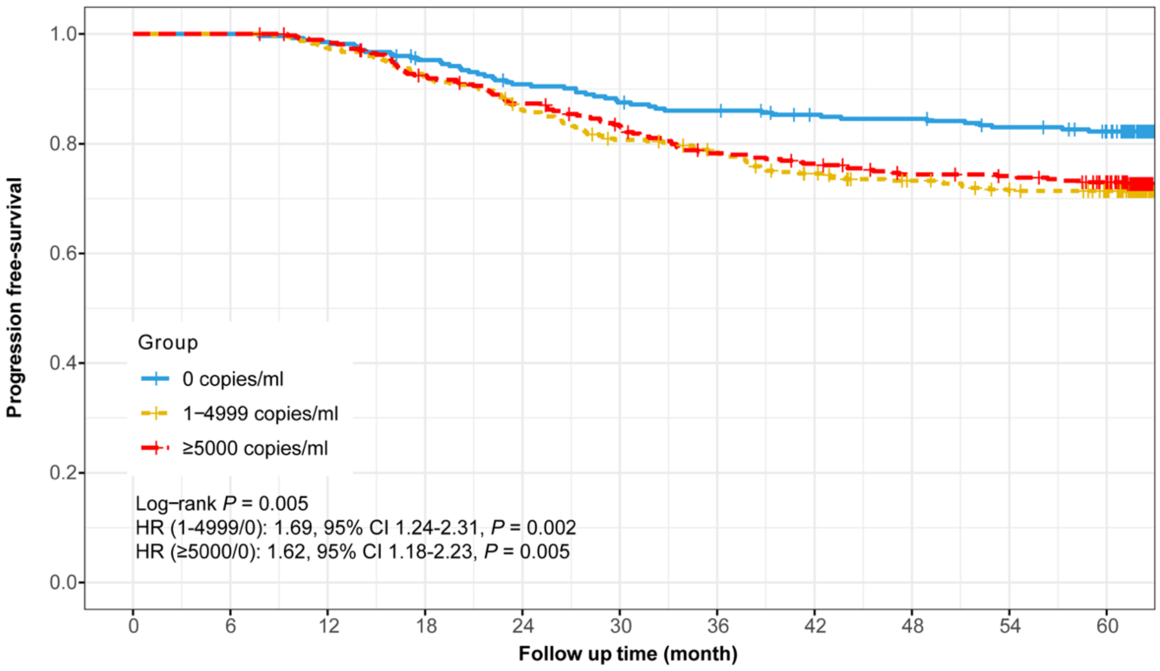

Number at risk

0	275	275	271	259	246	237	232	225	223	217	209
1−4999	400	400	390	369	343	319	307	288	276	265	254
≥5000	375	375	367	342	322	304	284	276	265	262	249

C.

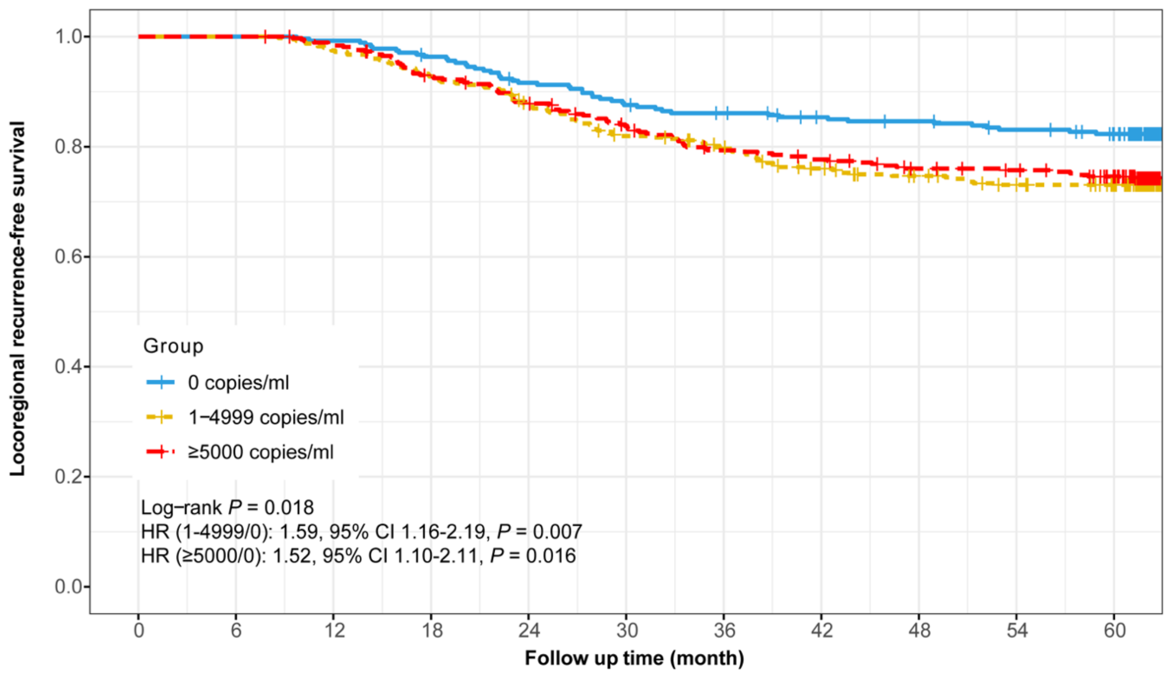

Number at risk

0	275	275	273	264	250	239	233	226	223	217	209
1−4999	400	400	390	371	347	323	311	290	278	267	255
≥5000	375	375	367	344	324	305	287	280	269	265	251

D.

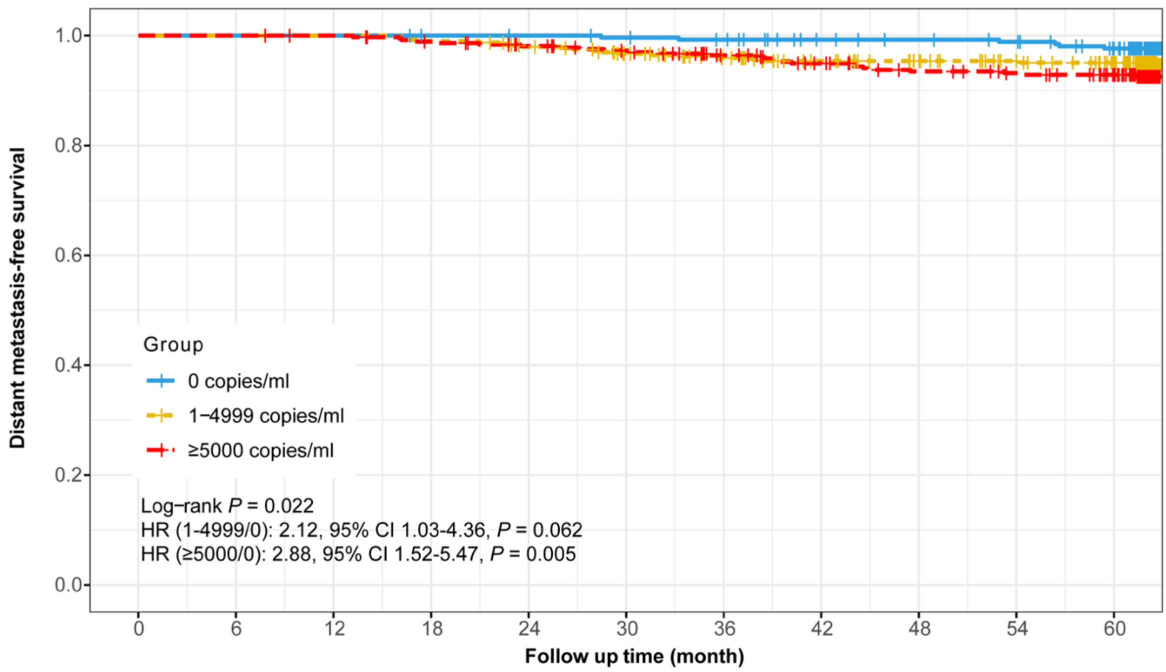

Number at risk

0	275	275	275	273	272	270	267	258	255	252	241
1−4999	400	400	400	396	387	373	359	344	332	320	301
≥5000	375	375	373	366	358	349	335	322	311	305	290

sFigure 4. Kaplan-Meier curves for overall survival (A), progression-free survival (B), locoregional recurrence-free survival (C) and distant metastasis-free survival (D) in all patients stratified by postradiotherapy plasma EBV DNA level (n=1050)

Abbreviations: EBV, Epstein-Barr virus; DNA, deoxyribonucleic acid

A.

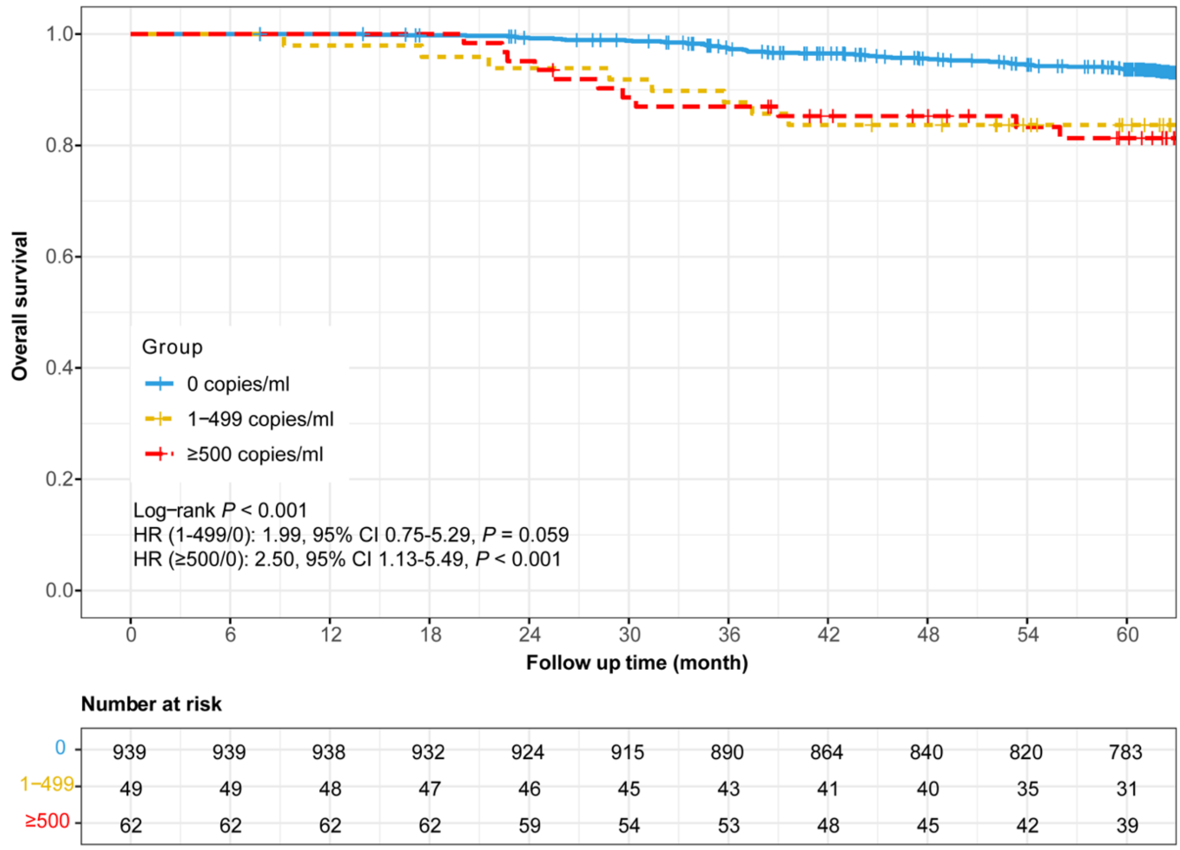

B.

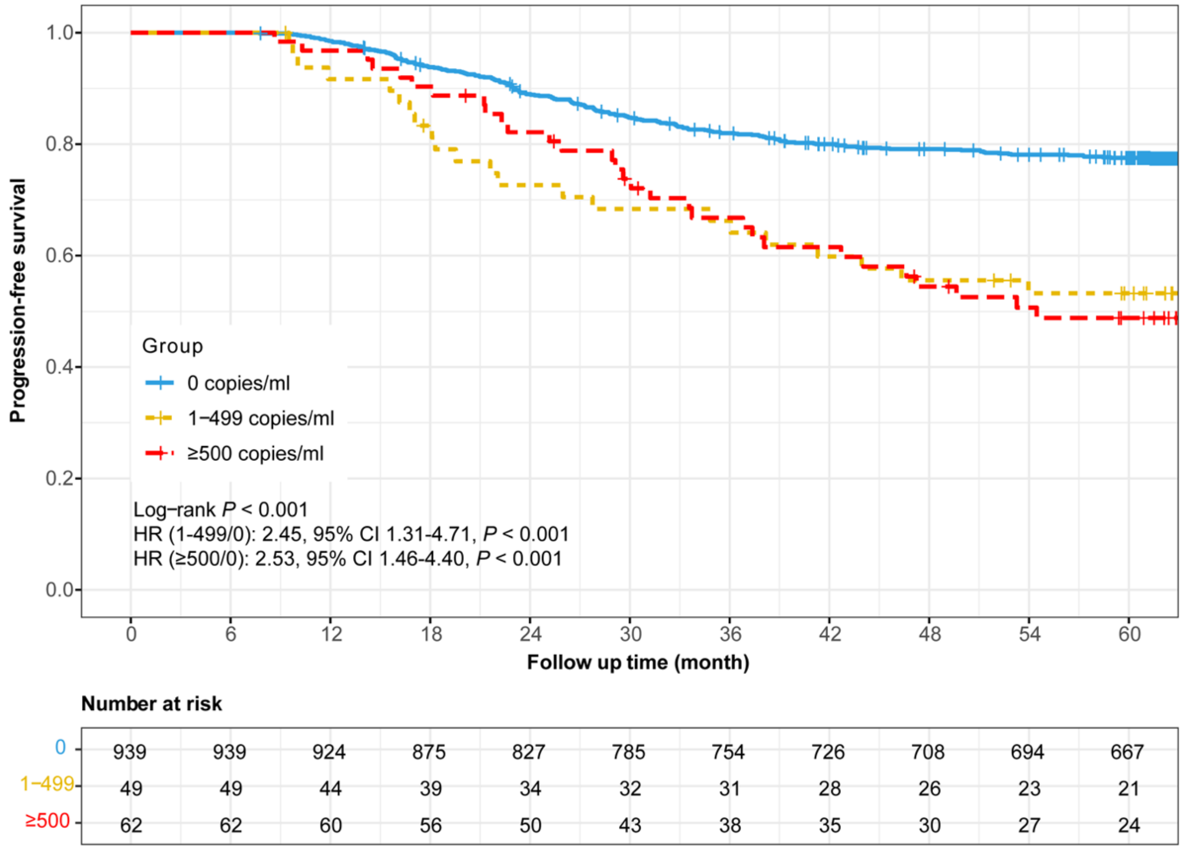

C.

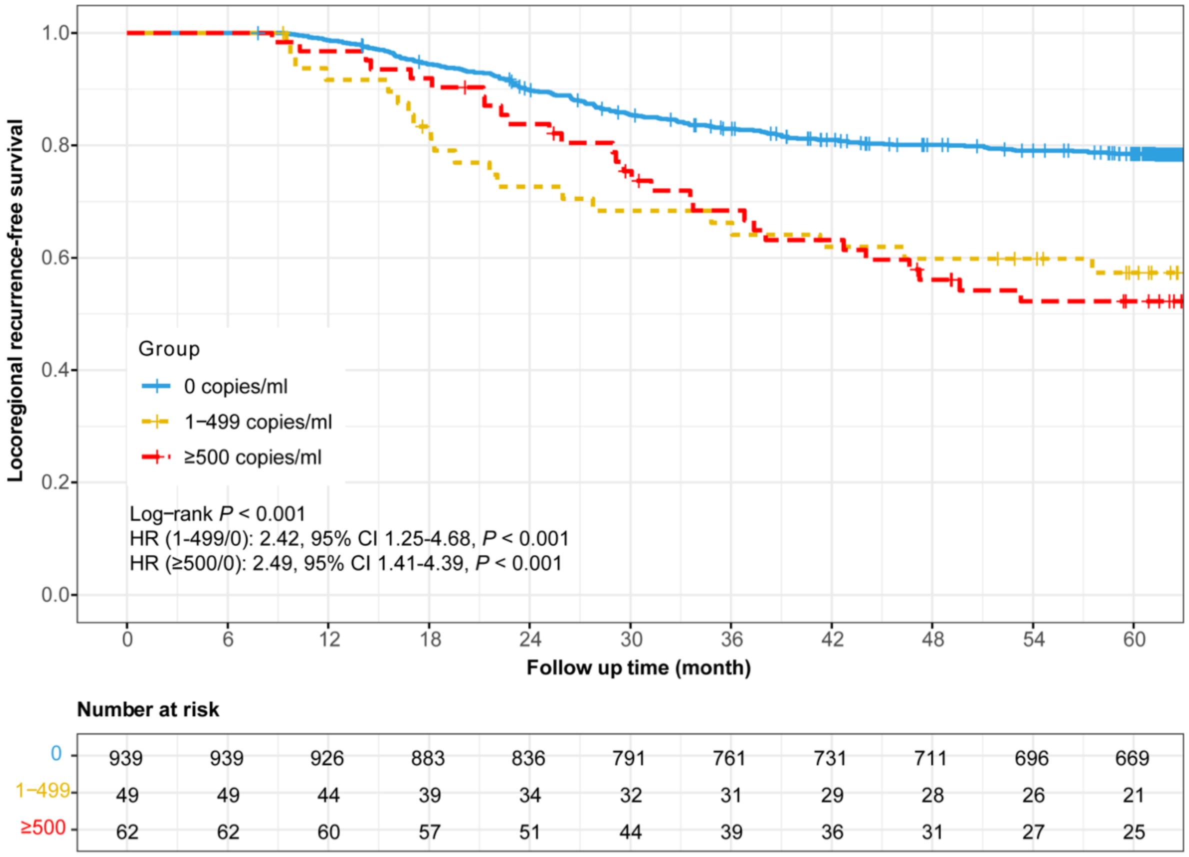
Locoregional recurrence-free survival
1.0
0.8
0.6
0.4
0.2
0.0
Group
0 copies/ml
1−499 copies/ml
≥500 copies/ml
Log−rank P < 0.001
HR (1-499/0): 2.42, 95% CI 1.25-4.68, P < 0.001
HR (≥500/0): 2.49, 95% CI 1.41-4.39, P < 0.001
0 6 12 18 24 30 36 42 48 54 60
Follow up time (month)
Number at risk
0 939 939 926 883 836 791 761 731 711 696 669
1−499 49 49 44 39 34 32 31 29 28 26 21
≥500 62 62 60 57 51 44 39 36 31 27 25

D.

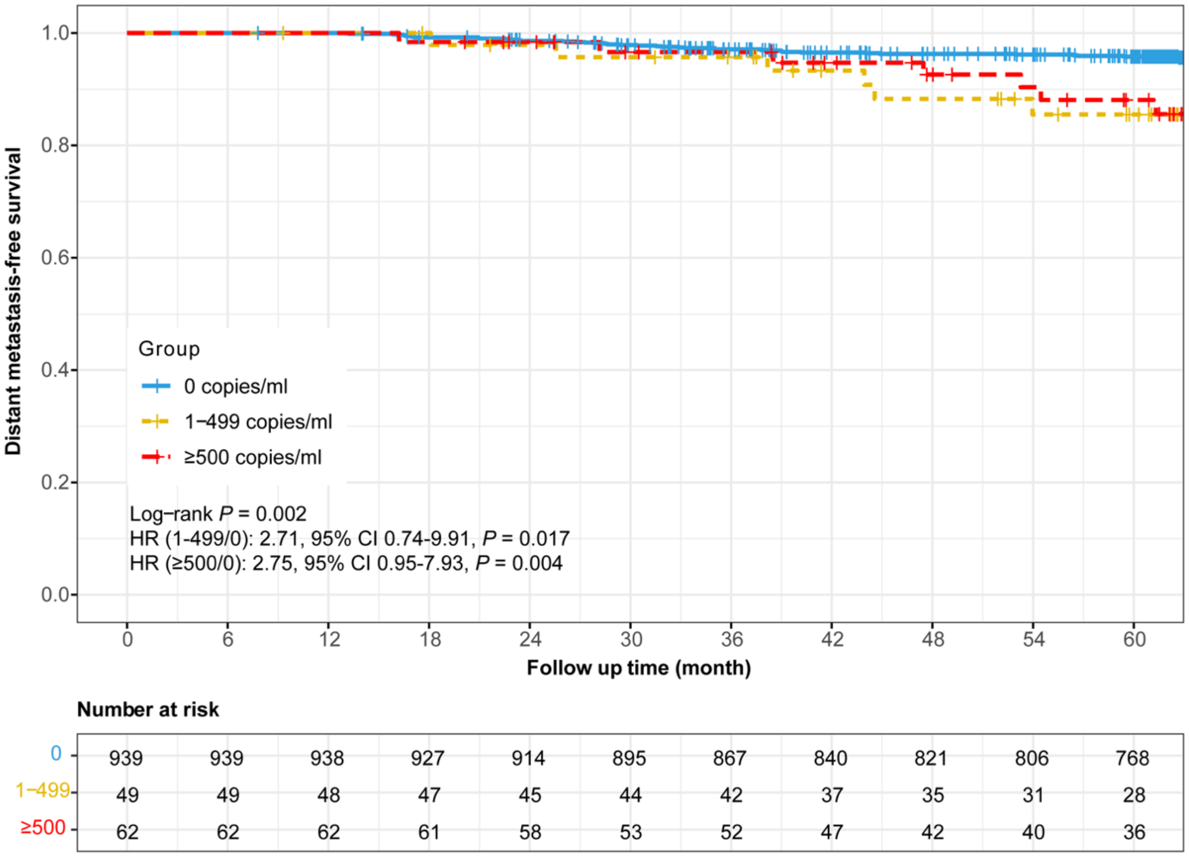
Distant metastasis-free survival
1.0
0.8
0.6
0.4
0.2
0.0
Group
0 copies/ml
1−499 copies/ml
≥500 copies/ml
Log−rank P = 0.002
HR (1-499/0): 2.71, 95% CI 0.74-9.91, P = 0.017
HR (≥500/0): 2.75, 95% CI 0.95-7.93, P = 0.004
0 6 12 18 24 30 36 42 48 54 60
Follow up time (month)
Number at risk
0 939 939 938 927 914 895 867 840 821 806 768
1−499 49 49 48 47 45 44 42 37 35 31 28
≥500 62 62 62 61 58 53 52 47 42 40 36

sFigure 5. Comparison of six models using area under the receiver operating characteristic curve calculated for the development cohort (n=736)

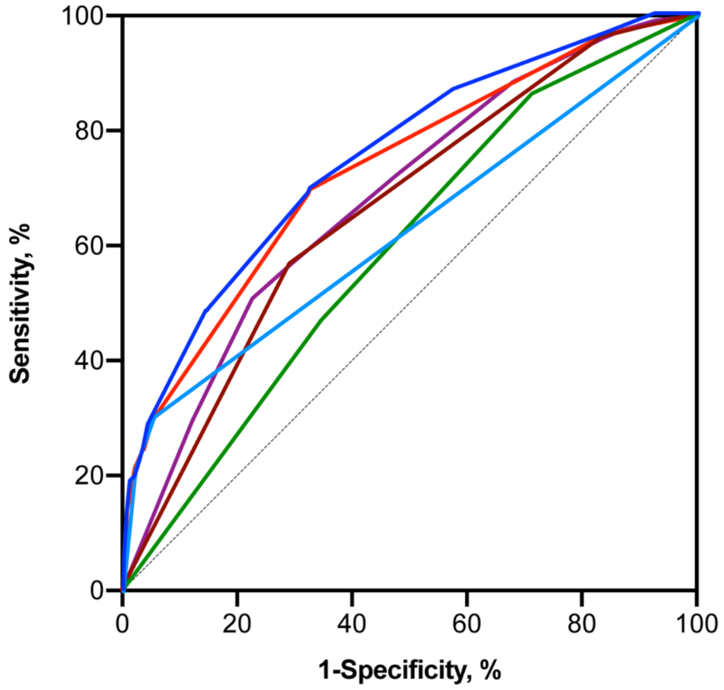

Model	AUC (95% CI)
Pretreatment EBV DNA	0.593 (0.541-0.646)
Postradiotherapy EBV DNA	0.627 (0.567-0.688)
Clinical stage	0.659 (0.607-0.710)
Clinical stage+Pretreatment EBV DNA	0.683 (0.633-0.733)
Clinical stage+Postradiotherapy EBV DNA	0.733 (0.682-0.783)
Prediction nomogram	0.752 (0.704-0.800)

sFigure 6. Kaplan-Meier curves for locoregional recurrence-free survival (A) and distant metastasis-free survival (B) in development cohort stratified by predicted risk in tumor residue (n=736)

A.

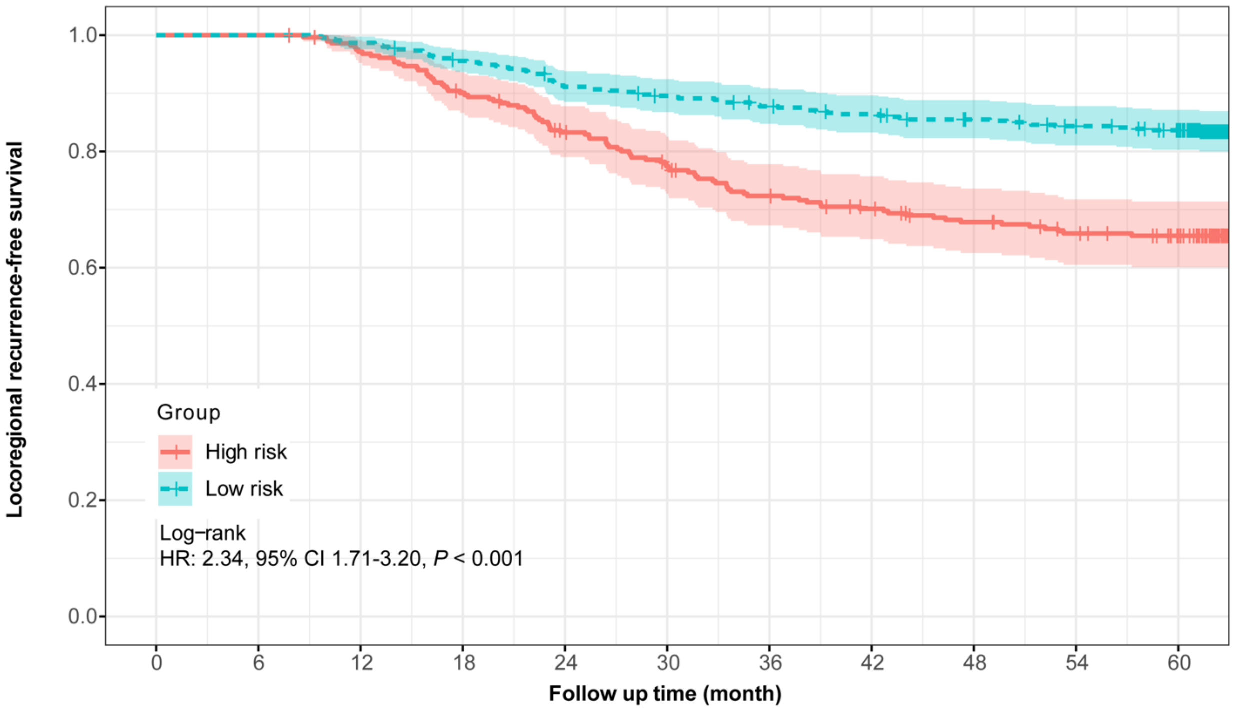

Number at risk

	0	6	12	18	24	30	36	42	48	54	60
High risk	284	284	274	253	231	213	197	187	177	168	155
Low risk	452	452	446	430	409	400	390	382	373	364	352

B.

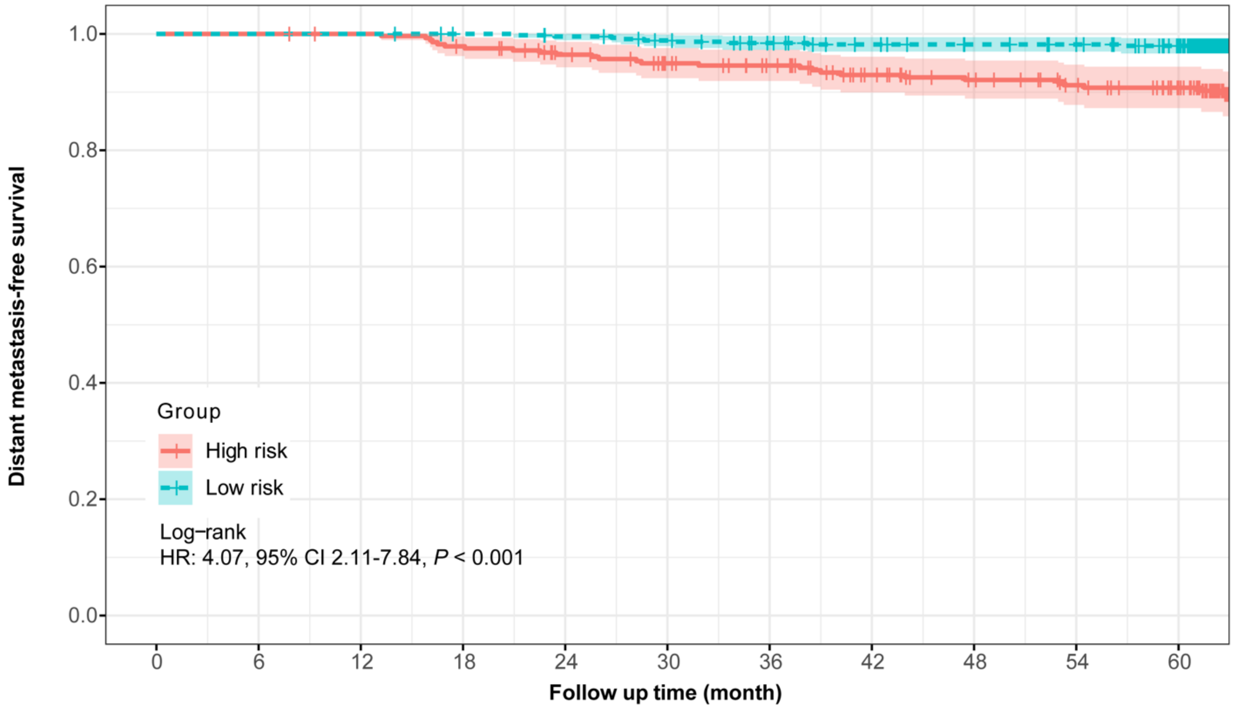

Number at risk

	0	6	12	18	24	30	36	42	48	54	60
High risk	284	284	282	275	263	251	242	223	212	201	184
Low risk	452	452	452	449	445	439	430	422	417	413	397

sFigure 7. Kaplan-Meier curves for locoregional recurrence-free survival (A) and distant metastasis-free survival (B) in validation cohort stratified by predicted risk in tumor residue (n=314)

A.

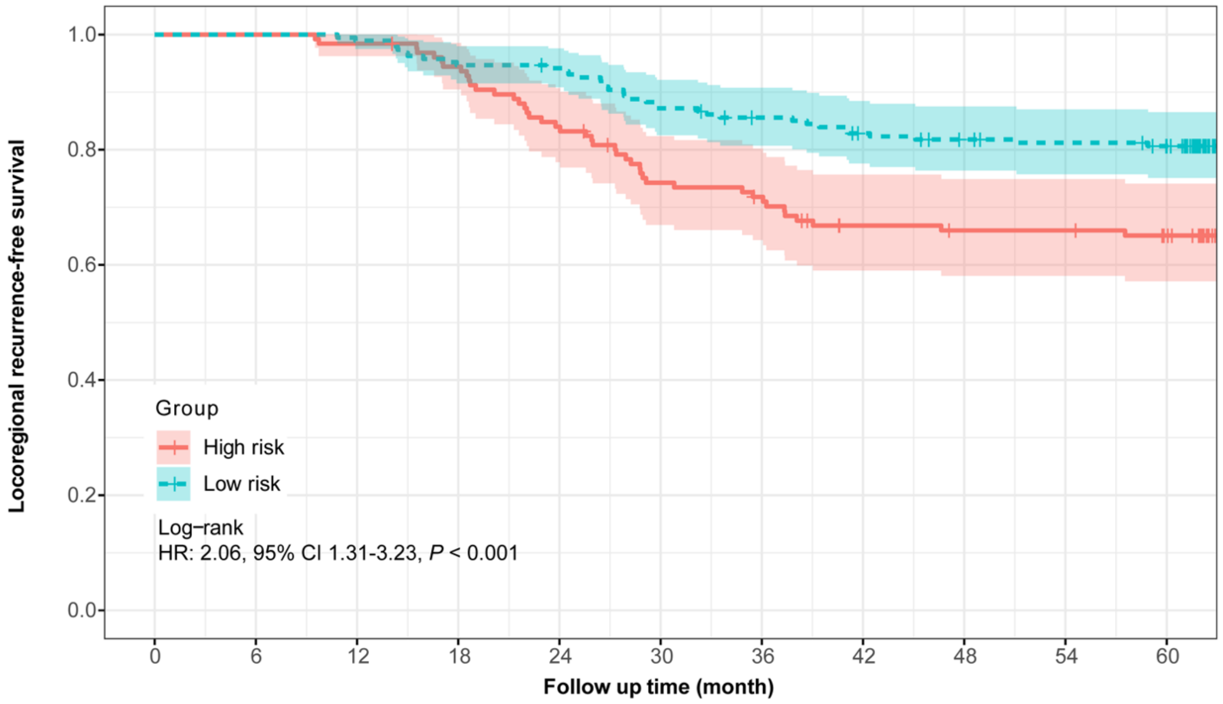

Number at risk

	0	6	12	18	24	30	36	42	48	54	60
High risk	126	126	124	118	105	91	87	77	75	75	71
Low risk	188	188	186	178	176	163	157	150	145	142	137

B.

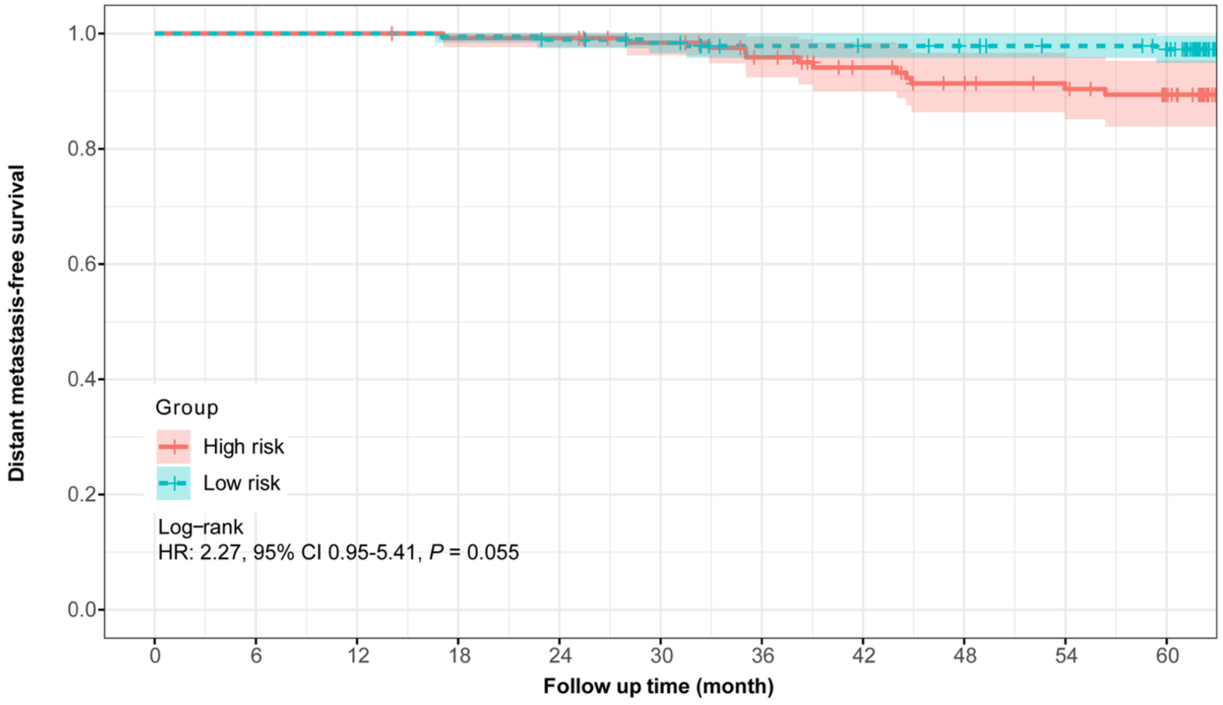

Number at risk

	0	6	12	18	24	30	36	42	48	54	60
High risk	126	126	126	124	124	120	113	104	96	93	87
Low risk	188	188	188	187	185	182	176	175	173	170	164

sFigure 8. Kaplan-Meier curves for overall survival (A), progression free-survival (B), locoregional recurrence-free survival (C) and distant metastasis-free survival (D) in patients with residue from development cohort stratified by predicted risk in tumor residue (n=122)

A.

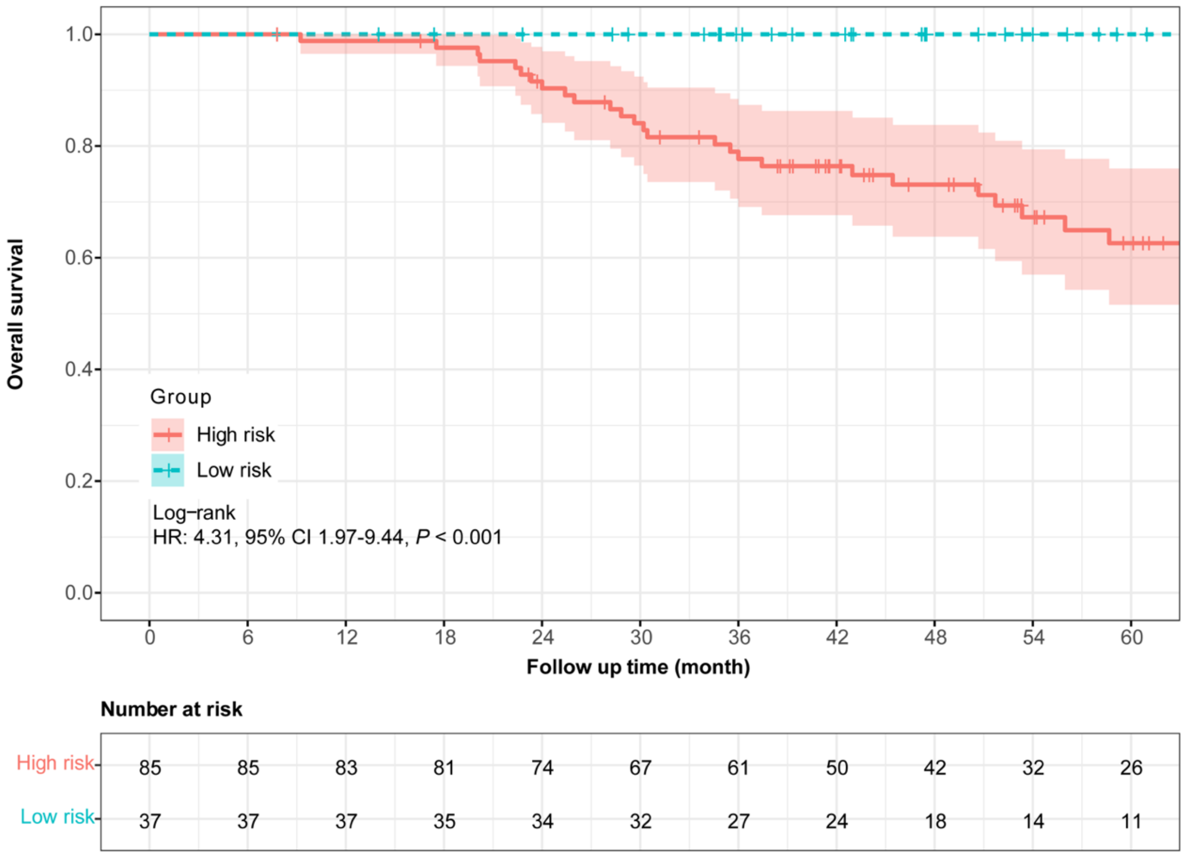

B.

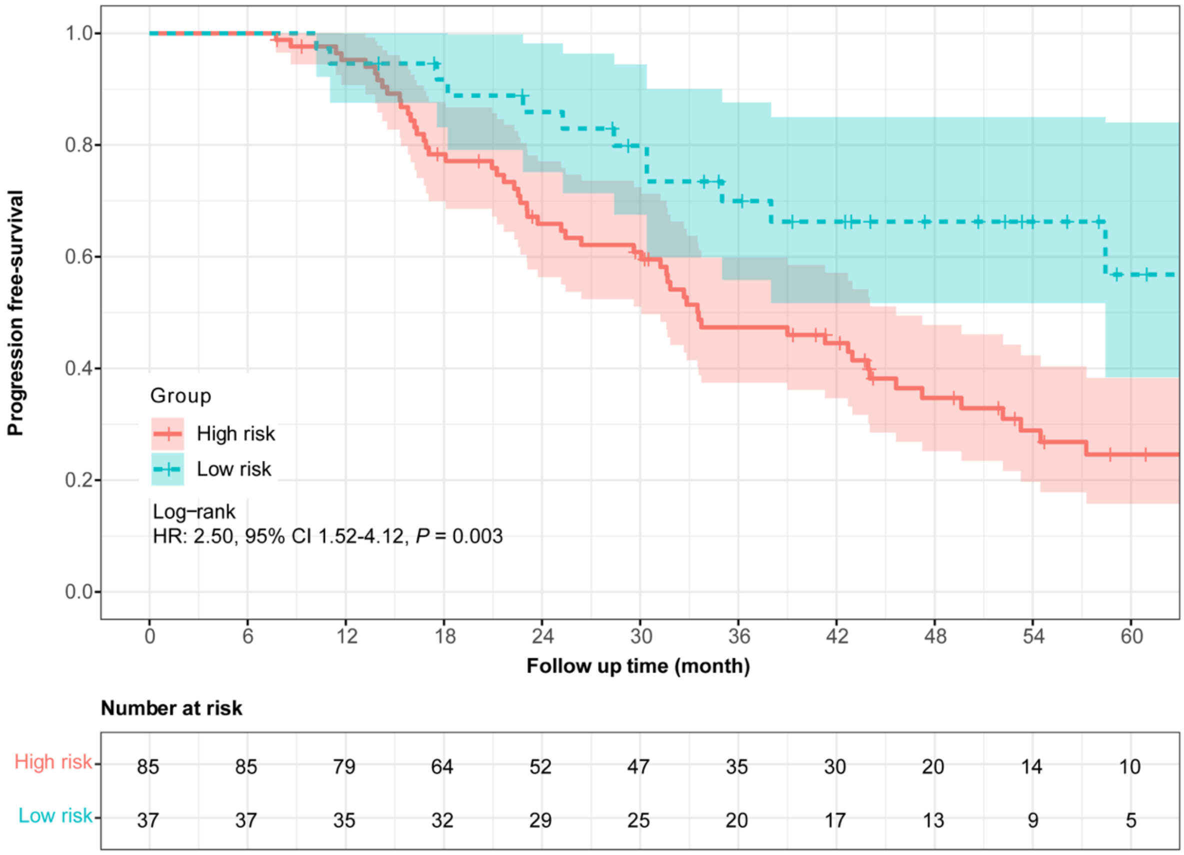

C.

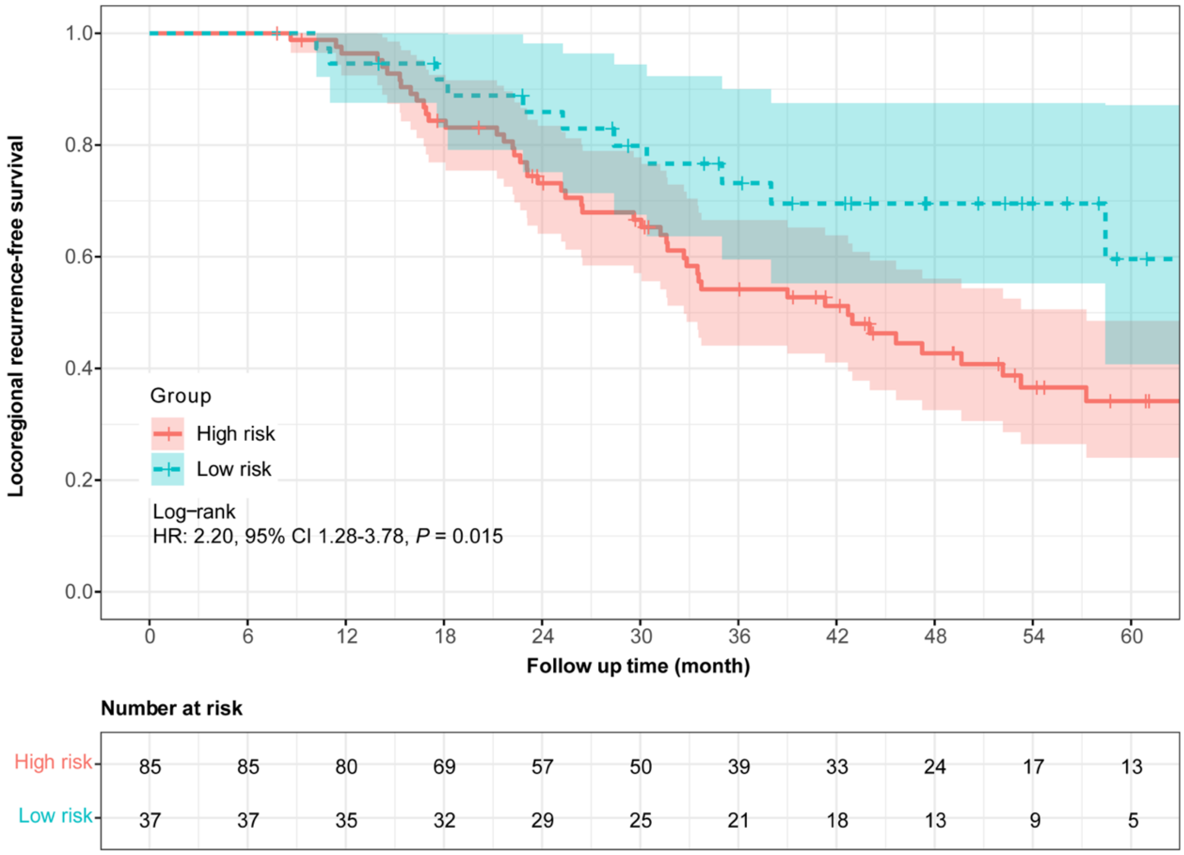
Locoregional recurrence-free survival
1.0
0.8
0.6
0.4
0.2
0.0
Group
High risk
Low risk
Log−rank
HR: 2.20, 95% CI 1.28-3.78, *P* = 0.015
0 6 12 18 24 30 36 42 48 54 60
Follow up time (month)
Number at risk
High risk 85 85 80 69 57 50 39 33 24 17 13
Low risk 37 37 35 32 29 25 21 18 13 9 5

D.

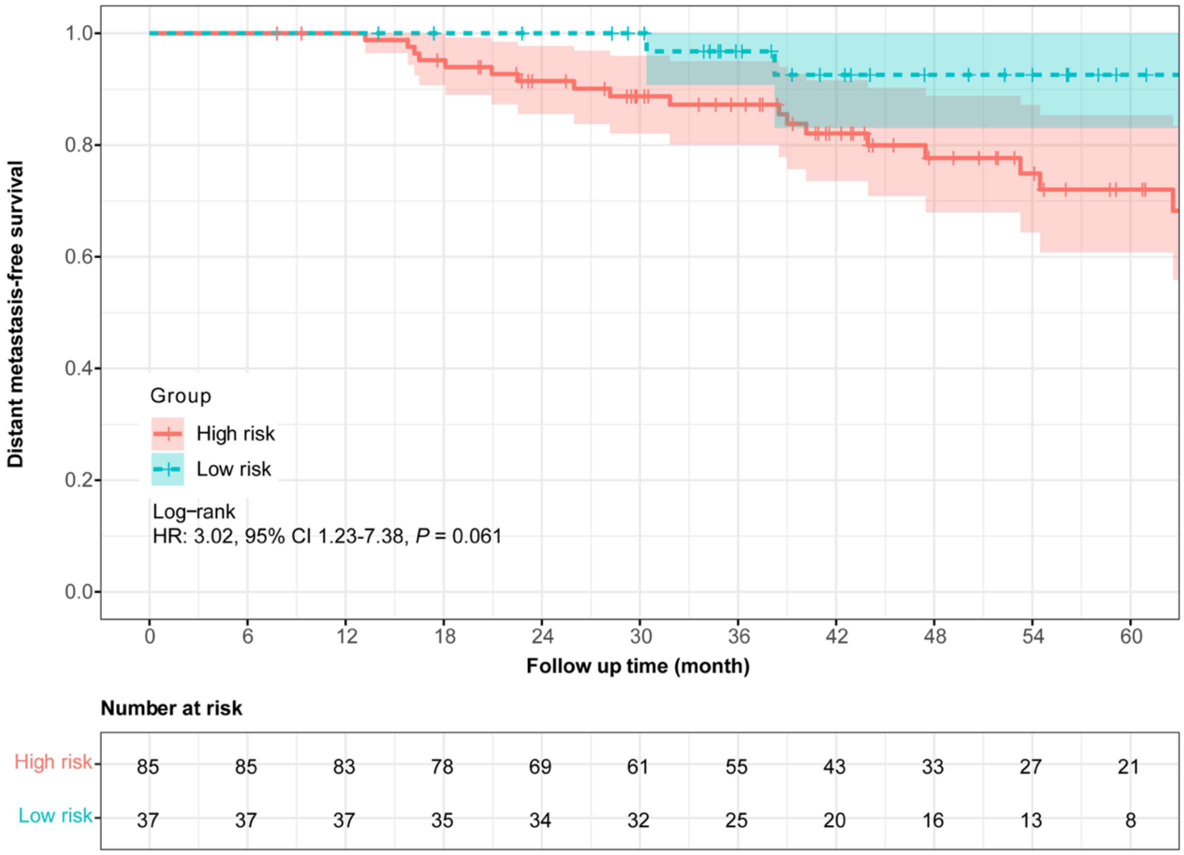
Distant metastasis-free survival
1.0
0.8
0.6
0.4
0.2
0.0
Group
High risk
Low risk
Log−rank
HR: 3.02, 95% CI 1.23-7.38, *P* = 0.061
0 6 12 18 24 30 36 42 48 54 60
Follow up time (month)
Number at risk
High risk 85 85 83 78 69 61 55 43 33 27 21
Low risk 37 37 37 35 34 32 25 20 16 13 8

sFigure 9. Kaplan-Meier curves for overall survival (A), progression free-survival (B), locoregional recurrence-free survival (C) and distant metastasis-free survival (D) in patients with residue from validation cohort stratified by predicted risk in tumor residue (n=68)

A.

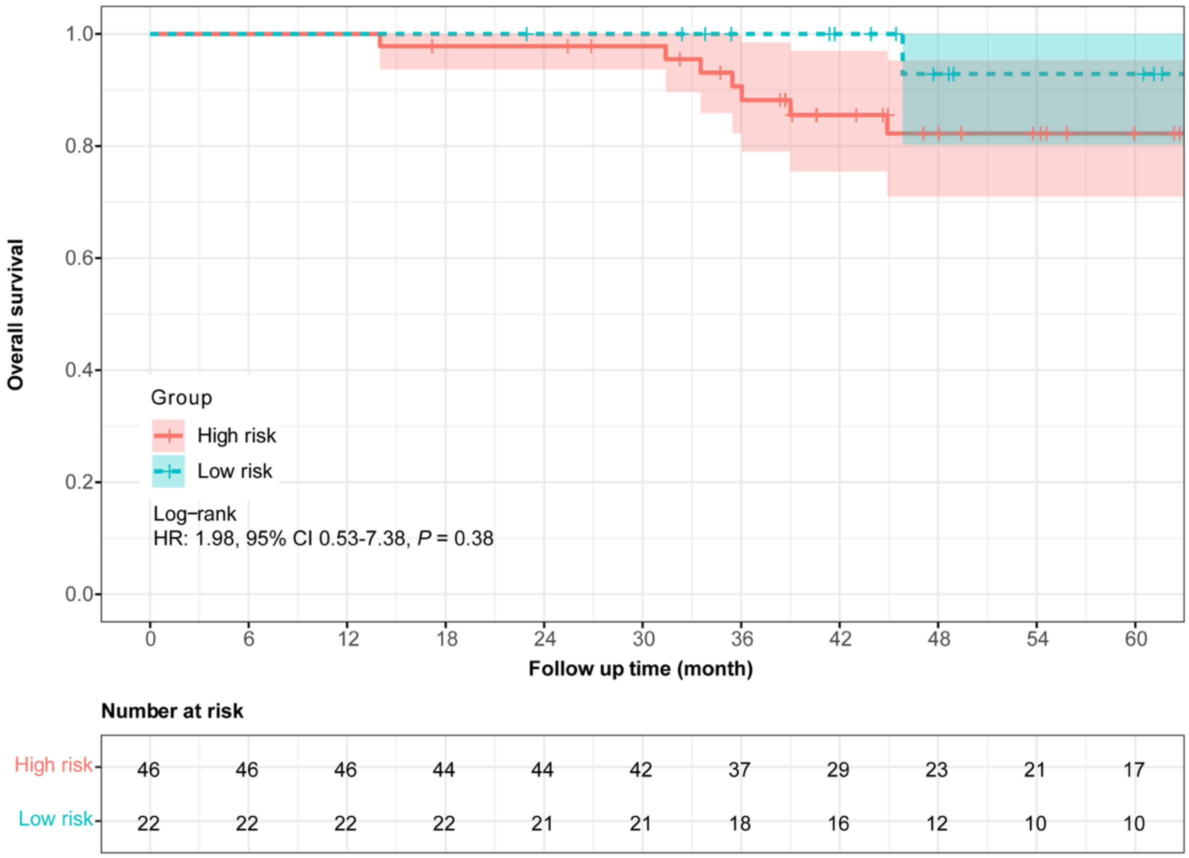

B.

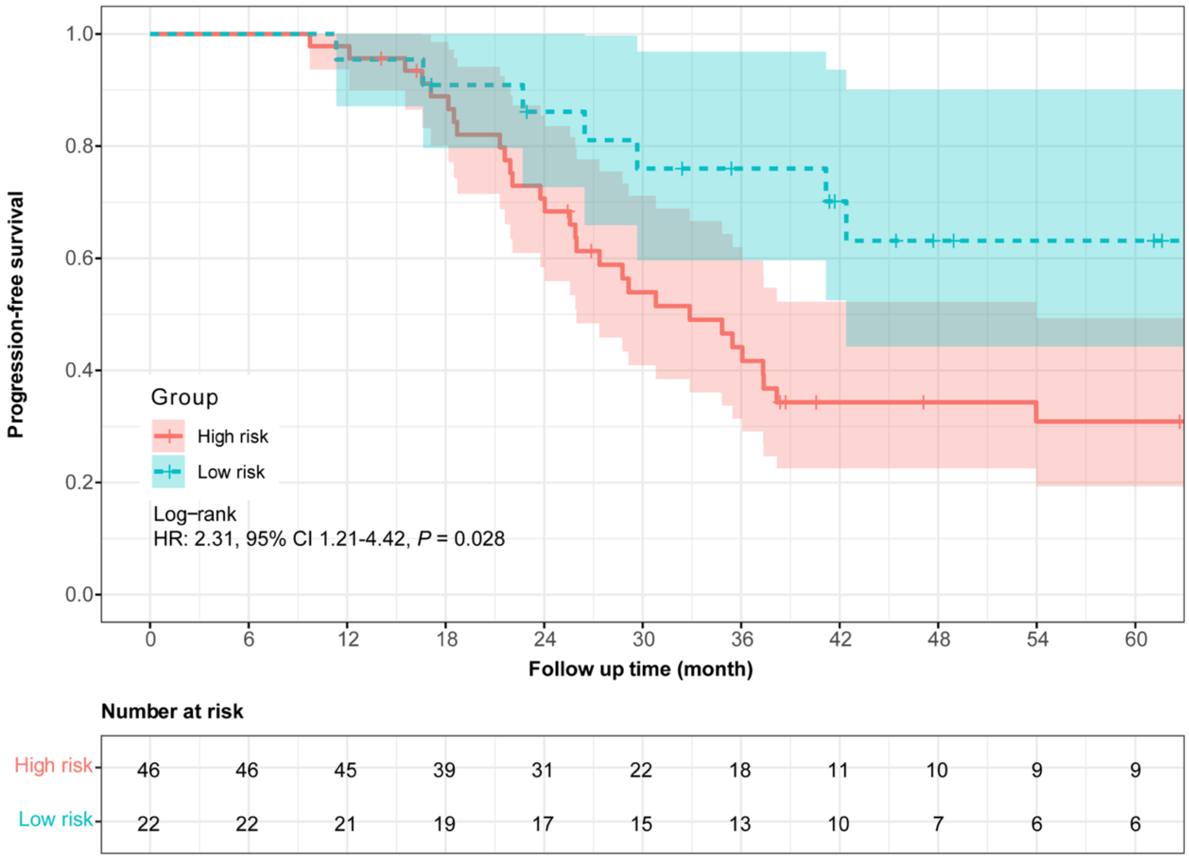

C.

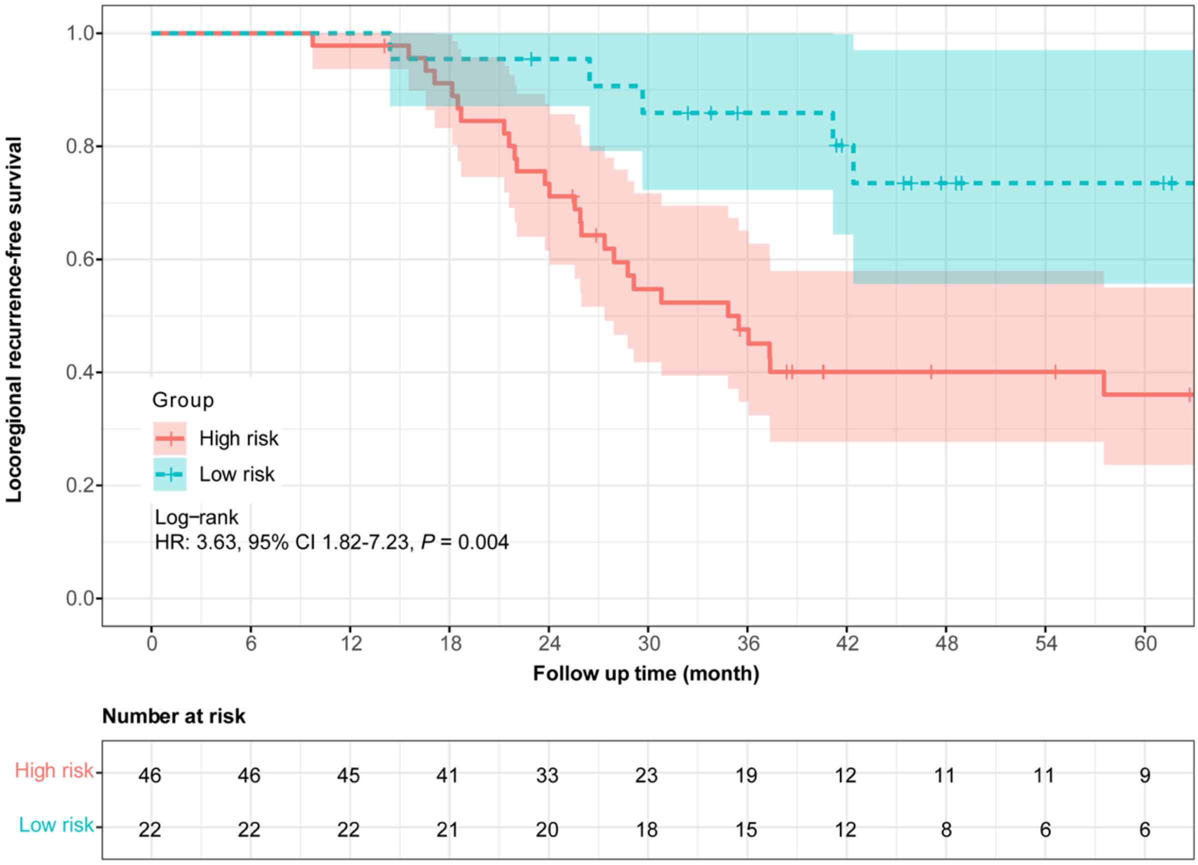
Locoregional recurrence-free survival
1.0
0.8
0.6
0.4
0.2
0.0
Group
High risk
Low risk
Log−rank
HR: 3.63, 95% CI 1.82-7.23, P = 0.004
0 6 12 18 24 30 36 42 48 54 60
Follow up time (month)
Number at risk
High risk 46 46 45 41 33 23 19 12 11 11 9
Low risk 22 22 22 21 20 18 15 12 8 6 6

D.

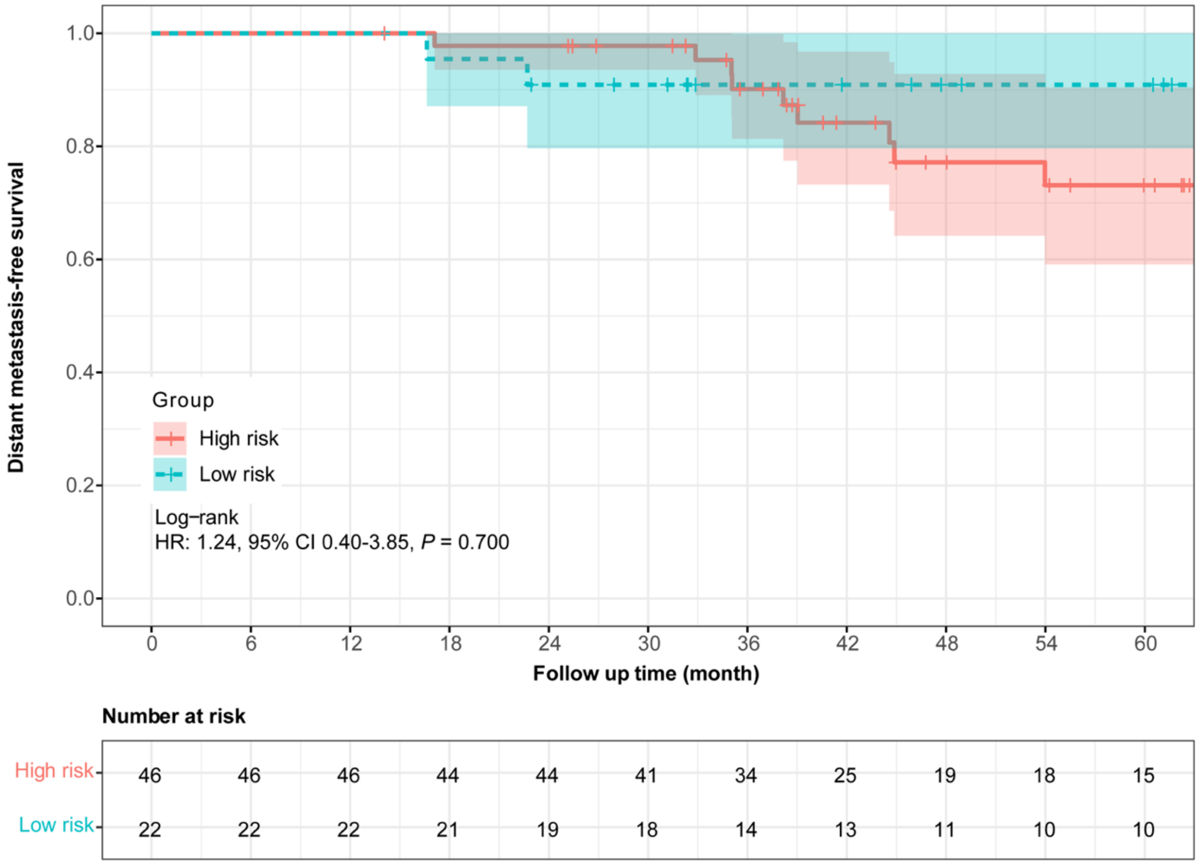
Distant metastasis-free survival
1.0
0.8
0.6
0.4
0.2
0.0
Group
High risk
Low risk
Log−rank
HR: 1.24, 95% CI 0.40-3.85, P = 0.700
0 6 12 18 24 30 36 42 48 54 60
Follow up time (month)
Number at risk
High risk 46 46 46 44 44 41 34 25 19 18 15
Low risk 22 22 22 21 19 18 14 13 11 10 10
